# SpaceGrow: efficient shape-based virtual screening of billion-sized combinatorial fragment spaces

**DOI:** 10.1007/s10822-024-00551-7

**Published:** 2024-03-17

**Authors:** Sophia M. N. Hönig, Florian Flachsenberg, Christiane Ehrt, Alexander Neumann, Robert Schmidt, Christian Lemmen, Matthias Rarey

**Affiliations:** 1 BioSolveIT, An der Ziegelei 79, 53757 Sankt Augustin, Germany; 2 Universität Hamburg, ZBH - Center for Bioinformatics, Albert-Einstein-Ring 8-10, 22761 Hamburg, Germany

**Keywords:** Chemical space, Ligand-based virtual screening, Structural superposition, Molecular shape, Fragment-based design, SpaceGrow

## Abstract

**Abstract:**

The growing size of make-on-demand chemical libraries is posing new challenges to cheminformatics. These ultra-large chemical libraries became too large for exhaustive enumeration. Using a combinatorial approach instead, the resource requirement scales approximately with the number of synthons instead of the number of molecules. This gives access to billions or trillions of compounds as so-called chemical spaces with moderate hardware and in a reasonable time frame. While extremely performant ligand-based 2D methods exist in this context, 3D methods still largely rely on exhaustive enumeration and therefore fail to apply. Here, we present SpaceGrow: a novel shape-based 3D approach for ligand-based virtual screening of billions of compounds within hours on a single CPU. Compared to a conventional superposition tool, SpaceGrow shows comparable pose reproduction capacity based on RMSD and superior ranking performance while being orders of magnitude faster. Result assessment of two differently sized subsets of the eXplore space reveals a higher probability of finding superior results in larger spaces highlighting the potential of searching in ultra-large spaces. Furthermore, the application of SpaceGrow in a drug discovery workflow was investigated in four examples involving G protein-coupled receptors (GPCRs) with the aim to identify compounds with similar binding capabilities and molecular novelty.

**Graphical abstract:**

SpaceGrow descriptor comparison for an example cut in the molecule of interest. Scoring scheme is implied for one fragment of this cut. 
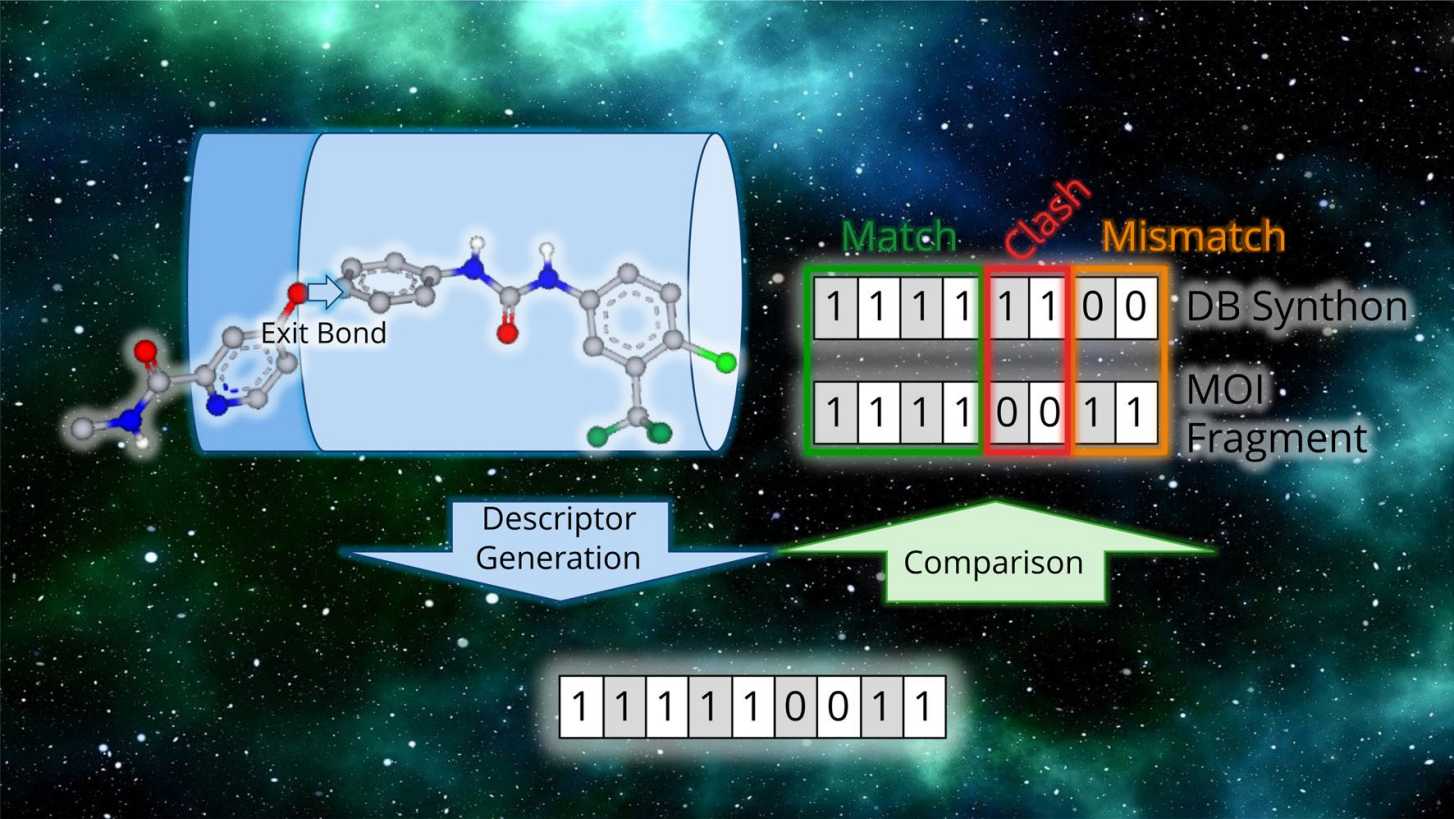

**Supplementary Information:**

The online version contains supplementary material available at 10.1007/s10822-024-00551-7.

## Introduction

Due to the growing interest in searching and analyzing giant make-on-demand chemical libraries, [1–4] novel cheminformatics approaches became indispensable. [5, 6] A navigation through reaction-driven combinatorial libraries, so-called chemical spaces, offers synthetically accessible compounds far beyond the reach of enumerable databases. [7] Given an active compound, the investigation of the proximal chemical space around it can accelerate the lead optimization process (SAR-by-Space). [8–11] The imminent interest in individual chemical spaces brought a huge variety of free, [12] proprietary, [7, 13–17] and make-on-demand [18–22] spaces as well as tools to create those. [23–25] While navigating such spaces using a variety of 2D methods is well established by now and incredibly fast, [26–29] comparable 3D methods are largely missing as of today. Yet, the ability to take molecular shape into account and thereby facilitate scaffold hopping is extremely desirable but more complex. [30–34] Make-on-demand libraries originate largely from combinatorial chemistry. Thus, using a combinatorial approach instead of an exhaustive enumeration of all possible synthesizable compounds comes with the benefit that the resource requirement scales approximately with the number of synthons instead of the number of all compounds. [31, 33] However, even the combinatorial way of a structure-based molecular docking routine for chemical spaces remains a task of heavy computational effort. [31–34]

In ligand-based design, small molecule superposition is a standard technique to assess 3D ligand similarity, to enable visual inspection, and to estimate the likelihood of a compound to be active. A huge variety of existing methods has been designed for diverse application scenarios. [35] Among established commercial superposition methods like ROCS, [36, 37] FlexS [38–40] or MOE, [41, 42] especially volume overlap between the query molecule and another compound was the main feature of the underlying scoring functions. While some approaches like ROCS have been tuned to significantly increase search speed, [43] these are still restricted to enumerated compound libraries. A recent, development parallel to ours shows an urgent need of a method to overcome these restrictions and to conquer 3D ligand-based searches in chemical spaces (Cheng C, Beroza P. Shape-Aware Synthon Search (SASS) for virtual screening of synthon-based chemical spaces. ChemRxiv. Cambridge: Cambridge Open Engage; 2023; This content is a preprint and has not been peer-reviewed.).

To tackle this problem, we developed a novel approach named SpaceGrow for shape-based 3D virtual screening of combinatorial chemical spaces containing billions of compounds within hours on a single CPU. Given a molecule of interest (MOI) as query, the method scores molecular overlays of built-up compounds conceived in the space. Thus, it does not require knowledge of the protein structure. The use of directional shape-based descriptors already showed its high potential in the structure-based design setting. [44, 45] In SpaceGrow, these descriptors enable a shape-based, fuzzy, and rapid search for similar and topologically-related molecules. Based on a comprehensive list of known drugs [21] and structures from PDBbind, [46, 47] a data set of 56 ligand pairs binding to the same binding site in homologous pockets was created. On this data set we compared SpaceGrow to the open-source superposition tool LS-align. [48] Pose ranking and retrieval of the MOI as well as the homologous ligand were evaluated. Additionally, 160 of the active conformations of known drugs were utilized to compare the quality of results found searching in the individually created eXplore S (containing 6x$10^4$ compounds) as well as the eXplore 2C (containing 6x$10^9$ compounds) subspaces. Finally, the potential of SpaceGrow to mine relevant chemistry for drug discovery campaigns was investigated. SpaceGrow was used as the starting point of a computational workflow to mimic the scenario of mining spaces for potential binders at four different G protein-coupled receptors (GPCRs). Ligand-target complexes were then evaluated by HYDE, [49, 50] a structure-based post-optimization and scoring procedure, for free-energy estimation. Results were further assessed for their Tanimoto similarity to the co-complexed ligand with the aim to identify candidates with molecular novelty.

## Methods

### Descriptor and database generation

An efficient shape comparison has already been successfully applied in structure-based design in the scenario of fragment growing. [45] The underlying method named FastGrow scores the match of a fragment shape with the shape of a binding pocket based on a specific type of descriptor. [44] The basic idea of the SpaceGrow approach is the reimplementation and adaption of the shape descriptor for an ultra fast ligand-based 3D screening of combinatorial chemical spaces. The algorithmic strategy of SpaceGrow and the theory of the adapted descriptor are further described in the following.

Given a molecule of interest (MOI) for the search of analogs in a combinatorial chemical space, consisting of fragments and connection rules representing reaction types. In the subsequent paragraphs, fragments used in the creation of the chemical space will be referred to as synthons to match the present terminological consensus.

As already mentioned, SpaceGrow searches in combinatorial spaces, not enumerated libraries. Chemical spaces are created from given building blocks and reaction rules by CoLibri, [51, 52] which stores the synthons as SMILES files and the reaction rules as FragmentSpaceFiles (FSF). Setting up the databases from the combinatorial spaces for using SpaceGrow was done with the FastGrowDBCreator. [53, 54]

For each synthon of the space, the bond at which the reaction attaches another synthon is refered to as the exit bond. The exit bond is considered as one directional vector pointing in the direction of the respective synthon volume.

To compare the MOI with the synthons of the space, the MOI is fragmented iteratively. Each bond at which the MOI is cut is used as the exit bond for the two resulting fragments. All acyclic bonds are dissected, but only one bond at a time. Therefore, the presented implementation of SpaceGrow leads to multiple two-fragment pairs for an MOI. Consequently, the current coverage extends to only two component reactions.

**Fig. 1:**
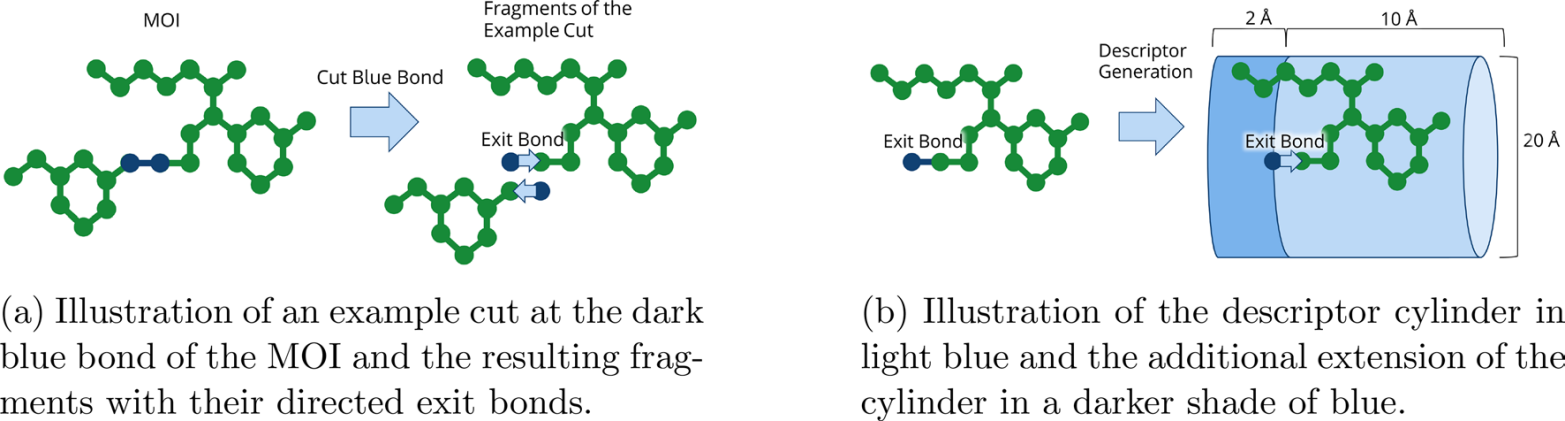
Descriptor generation in SpaceGrow. MOI fragmentation for an example cut is illustrated in (**a**) and a scheme of a descriptor for an MOI fragment or synthon is shown in (**b**)

To form the shape descriptor, a cylinder is constructed along the axis of the exit bond. For synthons, the cylinder begins at the atom of the exit bond at which another synthon is attached when the reaction forms a molecule. For fragments of the MOI, the cylinder is similarly aligned with the exit bond, i.e. the bond which was cut. An example of how an MOI might be cut at a bond is depicted in Fig. 1a. As suggested by Liu et al. [55] and Penner et al., [44] depth and radius of the cylinder were chosen to be 10 Å. The cylinder was also extended 2 Å into the opposite direction of the exit bond to more accurately describe fragments that extend beyond the start of the cylinder. This is sketched in Fig. 1b. The cylinder part in positive as well as the part in negative direction both are used for descriptor generation. The volume is sampled in regular distance increments of 1.5 Å along the cylinder axis. At each distance increment, rays are shot radially in a 20 ^∘^ pattern. In our implementation of the descriptor, rays are binned into intervals of 0.7 Å. If a line segment representing a bin intersects with the van der Waals sphere of an atom, its value is set to one. Otherwise it remains zero. This way, the rays describe the shape of the fragment by the volume of its atoms. A descriptor is build as a matrix composed of the bits representing these bins. Thus, the descriptor is named Ray Volume Matrix (RVM). [44] Within this matrix, rows represent the evaluated section at the axis of the exit bond while columns represent the angle at which the ray was sampled.

For a chemical space, the descriptors of 10 conformations per synthon are precomputed and stored in a database. To overcome the issue that fragments change when concatenated at the exit bonds, [56] geometry variants like induced planarity are stored with the fragments. For the fragments of the MOI, the descriptors and geometry variants are computed on the fly. The geometry variants are important for synthons whose atoms at the exit bond can adopt different atomic geometries. An example are synthons with an amino group at the exit bond: Depending on the type of reaction, the nitrogen can adopt a trigonal planar geometry in the reaction product (e.g., formation of an amide) or a tetrahedral geometry (e.g., formation of an aliphatic secondary amine).

### Descriptor comparison and pose scoring

**Fig. 2:**
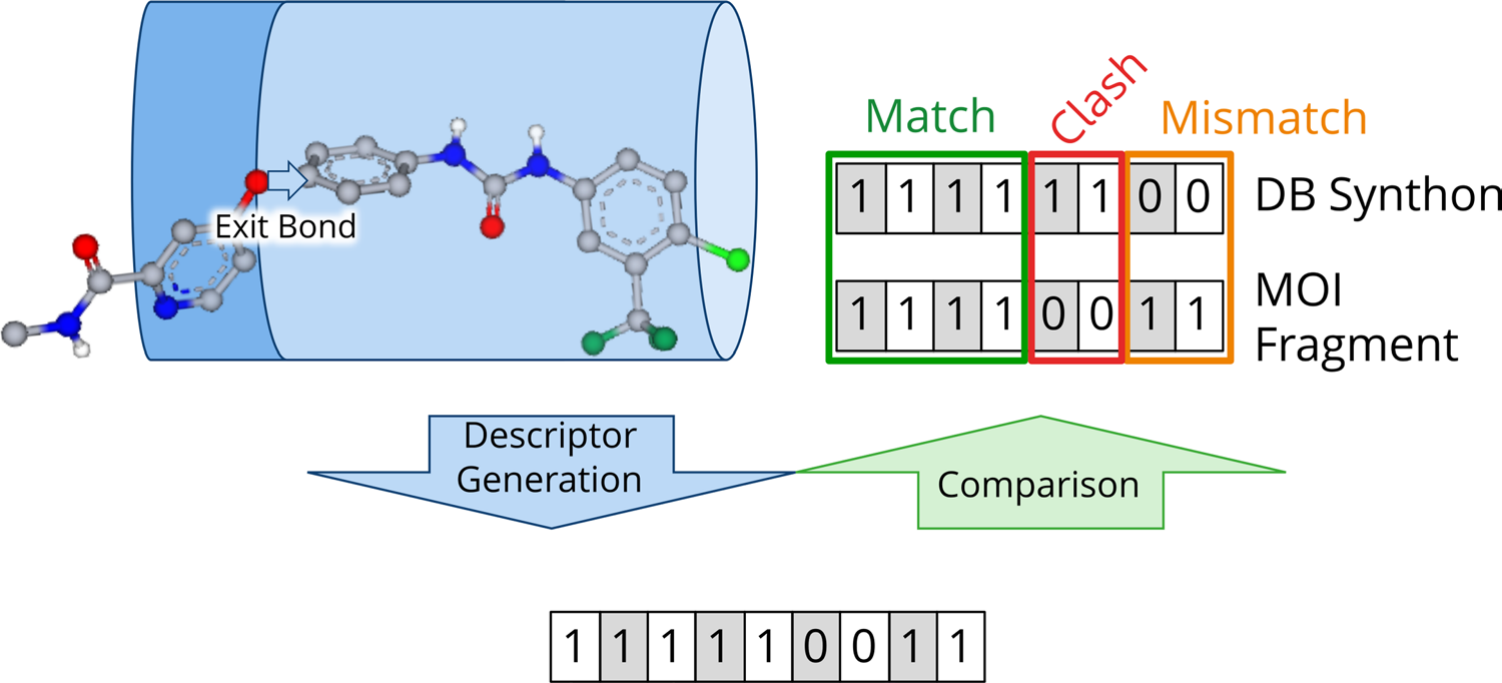
SpaceGrow descriptor comparison for an example cut in the MOI. Scoring scheme is implied for one fragment of this cut

Descriptors of two fragments can rapidly be compared by bit comparisons. Two equal bins at the same position give a match score. If the MOI fragment contains volume at a position, where the synthon does not, this is penalized as a mismatch. If the synthon contains volume where the MOI fragment does not, this is considered a clash, since we assume everything outside the MOI volume is potential protein volume. A clash is penalized twice as high as a mismatch. An example descriptor comparison is shown in Fig. 2.

The descriptors are translation invariant and partially rotation invariant with respect to the alignment of the axis given by the exit bond. The only degree of freedom left is the rotation along the axis of the exit bond. To find the ideal rotation at this axis, the synthon is rotated along the exit bond by applying bit shift operations on the RVM descriptor.

To find the best matching pair of synthons for the MOI, we iterate over all acyclic bonds of the MOI. In each iteration, the MOI is cut into two and the descriptors for both sides are generated. Both descriptors are scored independently against the descriptors for the synthons. For each synthon, the generated score, the cut bond of the MOI, the side of the MOI it was compared to, and the rotation with the best score are stored. After all acyclic bonds of the MOI were processed, the synthons are sorted in descending order by score and the top scoring combinations of compatible synthons are generated. Two synthons are compatible, if there is a corresponding reaction within the chemical space. Additionally, their scores need to come from MOI fragments that were the opposite parts of the same cut inside the molecule. Depending on the number of requested results, the synthons are combined until enough pairs are found in a result list, again sorted by score in descending order. If the length of the list reached the number of requested results and the combined score from the next considered synthons is not higher or equal to the score at the end of the result list, no better combinations can be found and the algorithm terminates.

Finally, the best scoring pairs of synthons are selected to generate the resulting molecules. For achieving a final molecular alignment, the fragments are mapped onto the respective part of the MOI by superposing the exit bonds. The torsion angle of the newly formed bond is derived from the descriptor comparison.

### Data generation for validation experiments

For an objective validation, a data set of overlays with a ground truth, i.e. ligands binding at an identical binding site, was generated. Starting with a published list of 3008 FDA approved drugs, [21] we searched in the PDBbind refined set (version 2020) [46, 47] for structures where these ligands were cocrystallized. With 160 ligand structures found in their respective target binding site, we conducted a SIENA [57] search. For this search, a binding site is defined by the sequence of amino acids that are located in a distance of 6.5 Å around the ligand. SIENA detects all binding sites with an identical sequence in homologous proteins from all PDBbind refined structures. Subsequently, the homologous protein structure is overlaid onto the structure of the query protein. Thereby, the ligands binding in the same active site are superimposed with respect to their native binding mode as ligand ensembles. Of each ensemble, we only kept the ligand of the homologous protein with the lowest backbone RMSD to the query structure. No cutoff for the backbone RMSD was defined. In some cases, no proper superposition was achieveable, thus leading to their exclusion from the validation set. Those cases include long fatty acids (e.g., PDB: 3UEV) or ligands with huge differences in their size (e.g.; PDB: 1V2J). The final data set contained 56 ligands pairs, with one reference and another binding ligand, in the following denoted as homologous ligand, both interacting at an identical binding site.

To make these ligands accessible for SpaceGrow as a chemical space, they were fragmented into a combinatorial chemical space by clipping all acyclic bonds. In the following, this chemical space will be called validation space. Resulting fragments with at least 25 % of the heavy atoms of the original ligand were kept. This resulted in 322 fragments from the 112 ligands of our data set. For four of the reference ligands and one of the homologous ligands no suitable fragments could be created. These ligands either did not contain an acyclic bond to cut or none of the created fragments met the 25 % criterion. Three reaction rules for the validation space were defined by the bond type at which the fragments were cut, i.e. the exit bond of the generated synthons may be a single, a double or a triple bond. When combining synthons at their exit bonds to generate compounds from the space, two synthons can only be combined if their exit bonds have the same bond type. To make all molecules of the validation space accessible for conventional superposition approaches, we enumerated all 34,134 molecules into a validation library.

Using the validation space and library, SpaceGrow was compared to the superposition tool LS-align. [48] LS-align was selected, since it was the only open-source superposition tool available with a mode and score that address the flexibility of the ligands and could be executed with the present hardware.

LS-align was executed with default settings with the following exceptions: The Flexi-LS-align method was enabled, and hydrogen atoms were considered. The ranking of the matches was realized based on the PC-score. The larger this score, the higher the rank of the target molecule. The flexible alignment of LS-align involves generating a list of rotatable single bonds and superposing the initial target conformer to the query using Rigid-LS-align. Well-aligned rotatable bonds are removed from the initial list of rotatable bonds. Next, three bonds whose rotation can change the conformation most are chosen to construct alternative conformers. Torsion angles are sampled between -180^∘^ and 180^∘^ in 60^∘^ steps. The best ten conformers are chosen for a Rigid-LS-align superposition to optimize the alignment.

With the ligand pairs binding in their native conformations, we generated a gold standard for two tests on SpaceGrow and LS-align. First, in a retrieval experiment we analyzed how good the tools were able to rank the reference ligand among all molecules from the validation space and library, respectively. Additionally, we investigated how good the tools were able to reproduce the binding mode of the bound pose by means of RMSD. In a second experiment, the ranking of the homologous ligands was evaluated when using the reference ligands as the MOI for a search. In order to remove bias from MOIs fragments in the validation space, all resulting molecules containing MOI fragments were omitted in the ranking of the homologous ligands. The RMSD of each homologous ligand from the superposition of the tools to its bound pose was compared as well. The second experiment shows particularly the capability of generating a reasonable pose with respect to the target protein of the MOI.

### Generation of eXplore subspaces and comparison 

To test SpaceGrow on individual chemical spaces of different sizes, we generated our own versions of the make-on-demand eXplore space. [21, 58] We decided on using the eXplore space since its reactions are publicly available. [59] The building blocks and reaction rules were provided by eMolecules. These were processed into the actual synthons in a suitable format for generating a SpaceGrow database using CoLibri. [51, 52] The sample of all two component reactions comprises about 6x$10^9$ molecules. We denote this space as the two component eXplore space, or short eXplore 2C. Given the fact that eXplore 2C was designed to be navigatable with SpaceGrow, it still contains approximately 6 billion compounds which makes the content too large for full enumeration. In order to prepare an enumerated library that can be processed with other superposition tools, we created a subset of the eXplore 2C space that contains 6x$10^4$ compounds, denoted as eXplore S. To generate this space, a random sample of the synthons from each reaction in the eXplore 2C was taken. Both spaces comprise three reaction types: amide coupling, substitution, and ring closing reactions. The eXplore S is composed as follows: 22,953 molecules from amide coupling, 6393 molecules from substitution reactions, and 29,148 molecules from ring closing reactions.

As a sanity check, searching conformers of molecules from the eXplore S led to the retrieval of about 70 % of these molecules among the top 10 SpaceGrow results (see Supplementary Information, Conformer Validation for details).

To elucidate potential blind spots of smaller libraries compared to vast combinatorial chemical spaces, results of SpaceGrow on the eXplore S and the eXplore 2C spaces are compared. For this purpose, the 160 structures of the FDA approved drugs [21] found in the PDBbind refined set (version 2020) [46, 47] were taken as MOIs to search in both spaces.

The main goal in structure-based virtual screening is to find molecules that display activity at a target of interest. In the ligand-based scenario on the other hand, similarity measures and scores allow no knowledge about a potential target but try to abstract information about the binding pocket from a given MOI. Therefore, additionally to the structure-agnostic approach, we investigated the results with an orthogonal method, i.e. a post-scoring with the MOI binding site was performed.

Here, the affinity and ligand efficiency of the found molecules was estimated using HYDE. [49, 50, 60] Based on a scoring function for affinity assessment in the structure-based design scenario considering hydrogen bond and dehydration energies, compounds can be locally optimized to best possibly fit the protein pocket.

### Mining for potential binders

 Over the past years, GPCRs remained difficult targets of highest interest [61]. Due to their high flexibility and anchoring in the phosphilipid bilayer, they represent difficult objectives for crystallization and the associated structure elucidation. The lack of 3D information for the target of interest coupled with challenging prediction of the binding site topology and the subsequent generation of ligand poses, impedes structure-based campaigns aiming at GPCRs. Ligand-based approaches therefore represent a viable alternative for mining potential binders without exhaustive calculations and refinement of ligand-GPCR complexes. For four members of the GPCR family we performed a SpaceGrow search in the eXplore 2C with the aim to identify compounds with similar or improved estimated binding affinity and molecular novelty. For this, structures of the GPCRs were downloaded from GPCRdb [62, 63] and prepared by removing crystalline water and non-ligand heteroatom groups.

To guide our shape-based search through chemical space, we utilized SpaceGrow to generate thousand results per target. These results were then scored and optimized by HYDE. [60] To efficiently scan for novel and potentially binding compounds with a low 2D similarity to the co-crystallized reference ligand, the result set was filtered. Only Compounds with a higher predicted affinity as the query molecule and a Tanimoto similarity of less than 0.7 were kept. The Tanimoto similarity was calculated using the Morgan Fingerprint implementation with radius four from RDKit. [64] The first ten results of this list were taken into further consideration for a more detailed analysis.

## Results and discussion

### Validation on active binding poses of known drugs and their analogs

 On the validation space of the 112 fragmented MOIs and corresponding homologous ligands, SpaceGrow is evaluated. The conventional superposition tool LS-align [48] is evaluated on the corresponding validation library, containing the 34,134 molecules enumerated from validation space. Here, the results of SpaceGrow and LS-align are compared in their ranking and pose reproduction by RMSD.

**Fig. 3:**
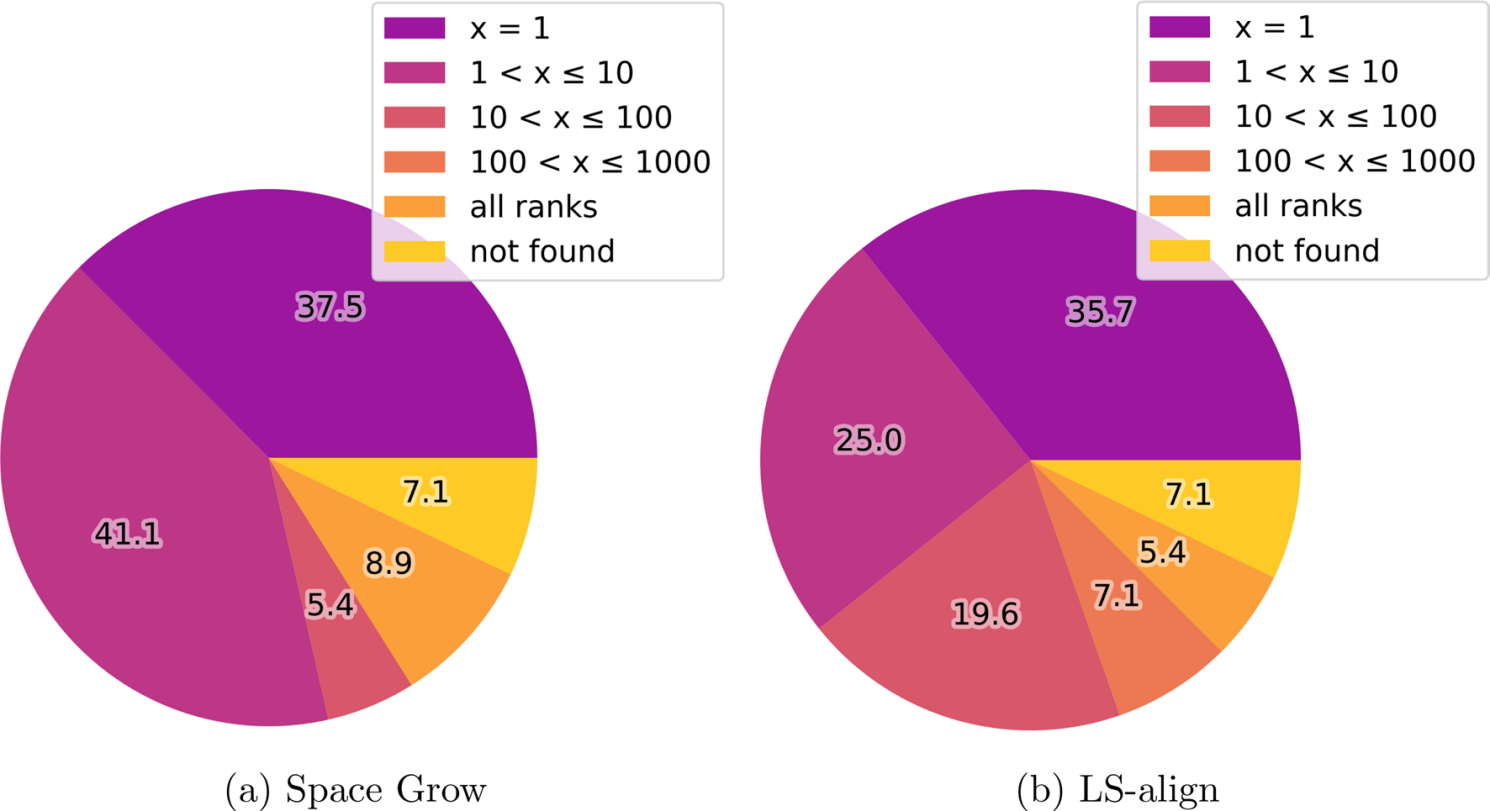
Retrieval of the 56 MOIs with an active binding pose derived from a PDBbind structure. Rankings are compared between the different tools. Overall, 34,134 molecules were ranked. The numbers of the pie charts give the percent of MOIs placed on a respective rank

Both, SpaceGrow and LS-align found at least 35 % of the 56 MOIs on first rank. While SpaceGrow found almost 80 % of the MOIs among the first ten ranks, here, LS-align was able to retrieve about 60 %. There were four MOIs (7.1 %), for which no fragments were present in the generated space. Thus, it was impossible for both tools to find these. Figure 3 shows the ranking of the MOIs in their active conformation by SpaceGrow and LS-align.

SpaceGrow poses had a median RMSD of 0.5 Å and a mean RMSD of 0.7 Å. LS-align reproduced the MOI pose with a median RMSD of 0.6 Å and a mean RMSD of 1.2 Å. Note that the conformations generated by SpaceGrow result from ten conformers per synthon pre-generated from the synthons’ SMILES strings for the SpaceGrow database. The respective RMSD values are depicted in Fig. 5.

**Fig. 4:**
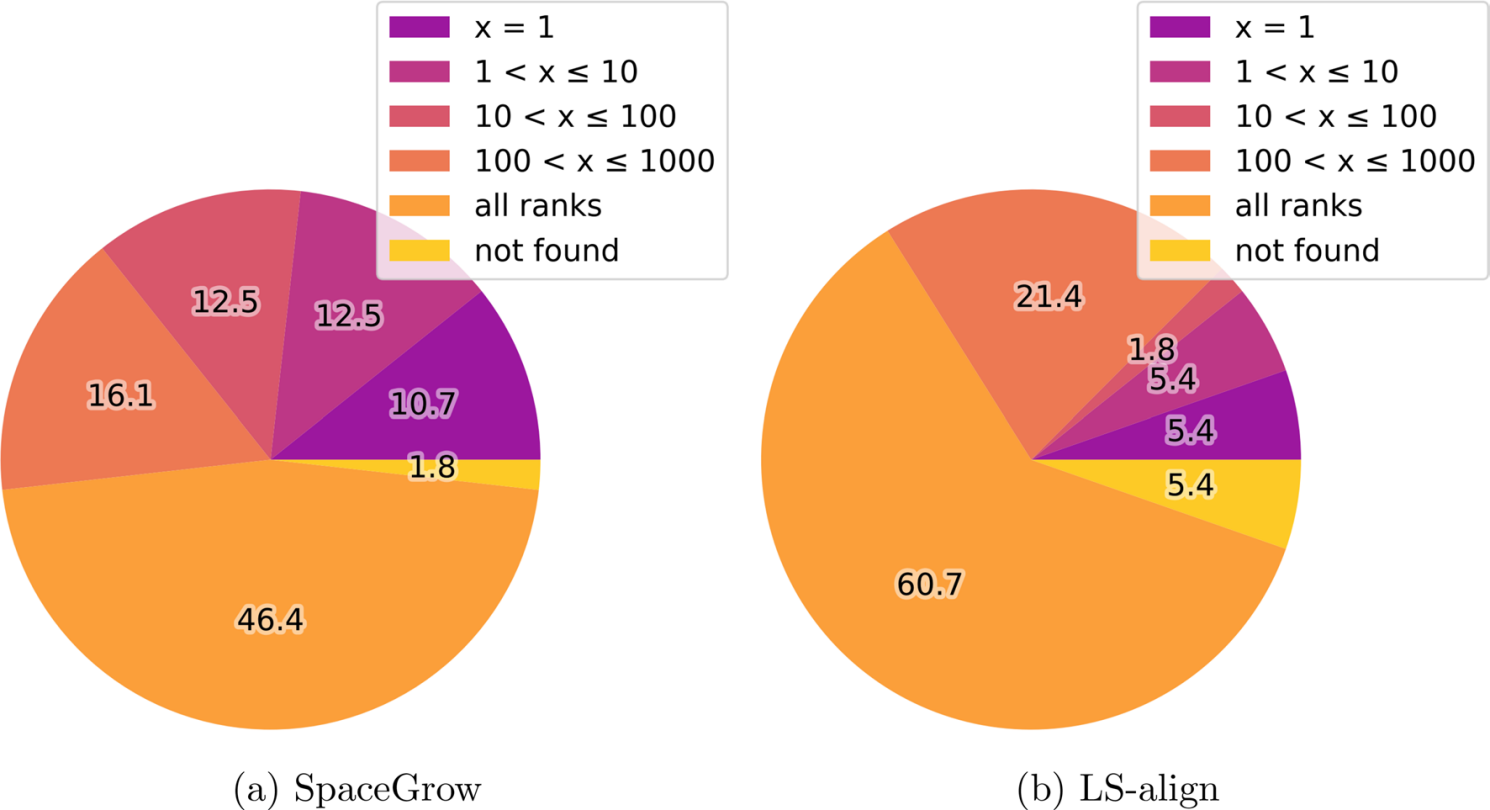
Ranking of homologous ligands for the 56 MOIs with an active binding pose derived from a PDBbind structure. Rankings are compared between the two tools. Overall, 34,134 molecules were ranked. The numbers of the pie charts give the percent of molecules placed on a respective rank

For both tools, ranking and reproduction for the homologous ligands of the MOIs was much more difficult than for the MOIs themselves as Fig. 4 indicates. While SpaceGrow still found more than 20 % of the homologous ligands among the first ten ranks, LS-align found about 10 %. In contrast to SpaceGrow, for some molecules of the validation library LS-align was not able to generate poses. While one homologous ligand was not part of the space (1.8 %), LS-align was not able to find poses of three homologous ligands (5.4 %).

**Fig. 5:**
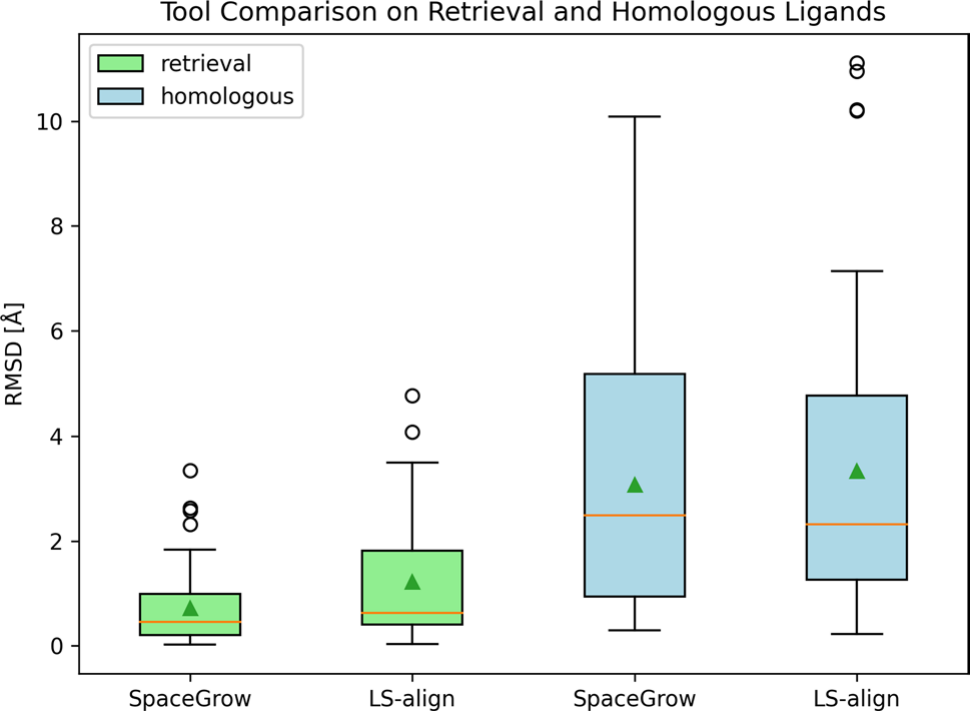
Comparison of RMSD values for the poses of the retrieved MOI (light green) and the homologous ligand (light blue) generated by the alignments of SpaceGrow and LS-align. The orange line indicates the median value. The green triangle marks the average value

As can be derived from Fig. 5, for both tools the mean and median RMSD values were distributed around 3 Å. Maximum values are reaching up to 10 Å.

Summing up, both benchmarking experiments were challenging for the evaluated tools. Figure 5 shows widely spread distributions, especially for the homologous ligands, indicating that the molecules inside the benchmark are diverse in their level of difficulty for all tested tools. The four most difficult ligand pairs for both tools were further analyzed in the Supplementary Information, Fig. 4. SpaceGrow outperforms LS-align in pose ranking for both, the MOI and the homologous ligands. Furthermore, SpaceGrow is superior in pose reproduction of the MOIs and shows slightly lower average and median RMSD values for the homologous ligands than LS-align. In summary, the investigated tools displayed the ability to rank and reproduce active poses of shape-related molecules. Shape-related molecules are resembled by our pairs of MOIs and their homologous ligands.

### Comparison of results from spaces of different size

 Since no ligand pairs but only the MOIs were needed for the following analysis, there were no more restrictions in picking the MOIs. Therefore, all 160 active MOI poses from mapping a list of known actives [21] against PDBbind [46, 47] were utilized. Analyzing the two eXplore subspaces eXplore S and eXplore 2C, it should be noted that the smaller space eXplore S was constructed to contain exactly the same reactions as the large space but with fewer synthons per building block.

**Fig. 6:**
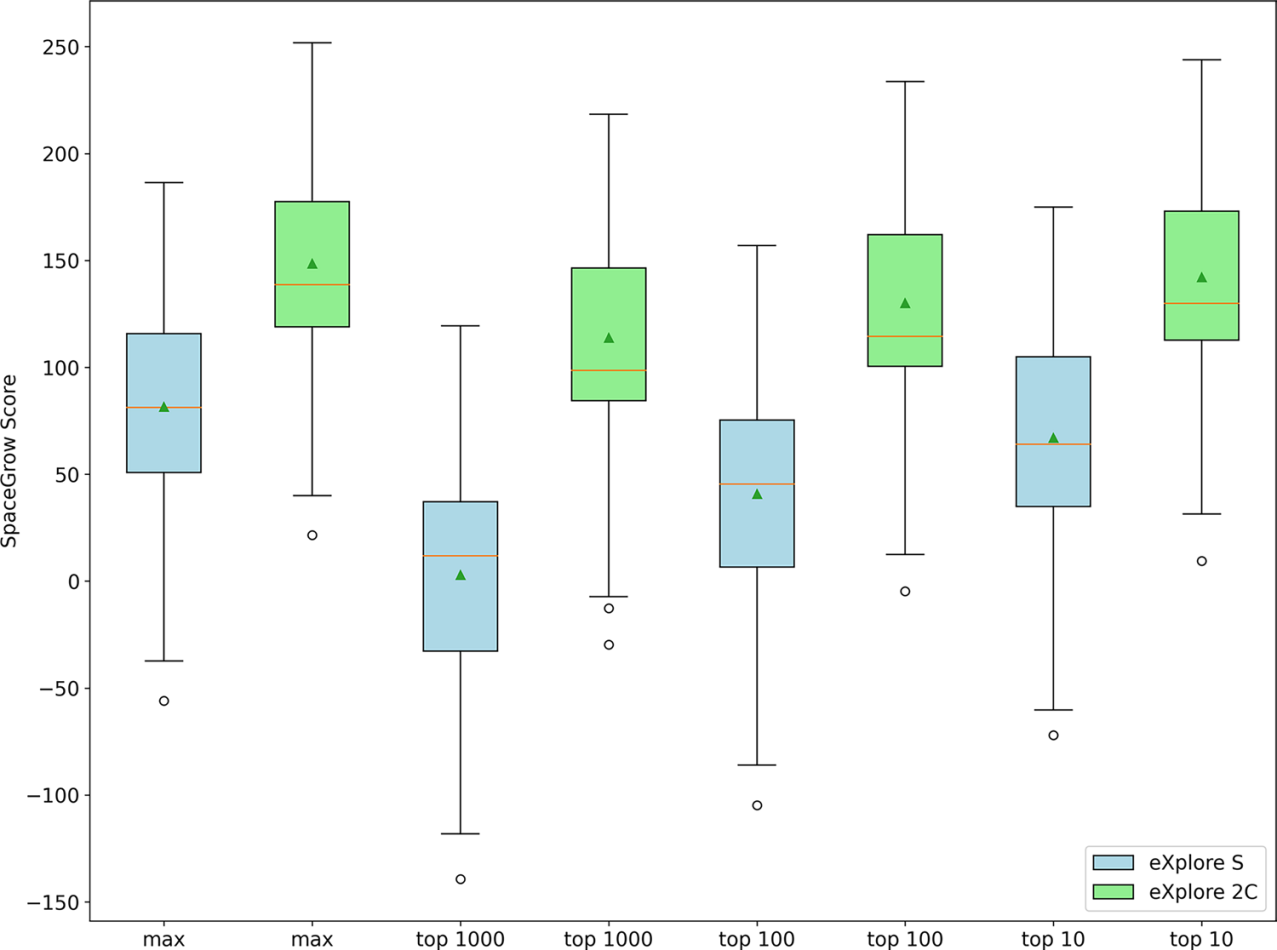
SpaceGrow Score distributions of the maximum score (max) and the median score of the top thousand, top hundred, and top ten results from the 160 searches in the eXplore S (blue) and eXplore 2C (green), respectively. The orange line marks the median value. The green triangle marks the average value

The two boxplots for the maximum SpaceGrow scores found during the search of each MOI in the eXplore S as well as the eXplore 2C are shown in Fig. 6. The average of the maximum scores over these 160 results improves by approximately a factor of two for the larger space results, implying better shape complementarity of the results.

Taking into account the median of the SpaceGrow score values over the top thousand results, the average over the 160 searches was over thirty times higher when searching in the larger space. A comparable observation was also made analyzing the median values of the top hundred and top ten results. This suggests that larger chemical spaces can offer more possibilities that can match the 3D profile of the query better.

The top thousand results from eXplore S were on average composed out of 620 different fragments (standard deviation: 72). The top thousand results from eXplore 2C were composed of 344 fragments on average (standard deviation: 148). A similar observation was made by counting the unique Bemis-Murcko scaffolds within the top thousand results for each MOI. Scaffolds were computed using RDKit. [64] Results found within eXplore S on average contained 213 unique scaffolds (standard deviation: 168) while results from eXplore 2C contained 142 unique scaffolds (standard deviation: 101). Histograms of the unique fragment and scaffold counts within the top hundred and top thousand results can be found in Fig. 5 of the Supplementary Information. Since the current version of SpaceGrow reports results by the best combined score without further clustering or filtering for diversity, this indicates that the larger space offers synthons with a very good shape match with the potential to form multiple interesting fragment combinations on top ranks. Thus, larger spaces offer the possibility to deeply explore SAR around fragments with high shape similarity.

**Fig. 7:**
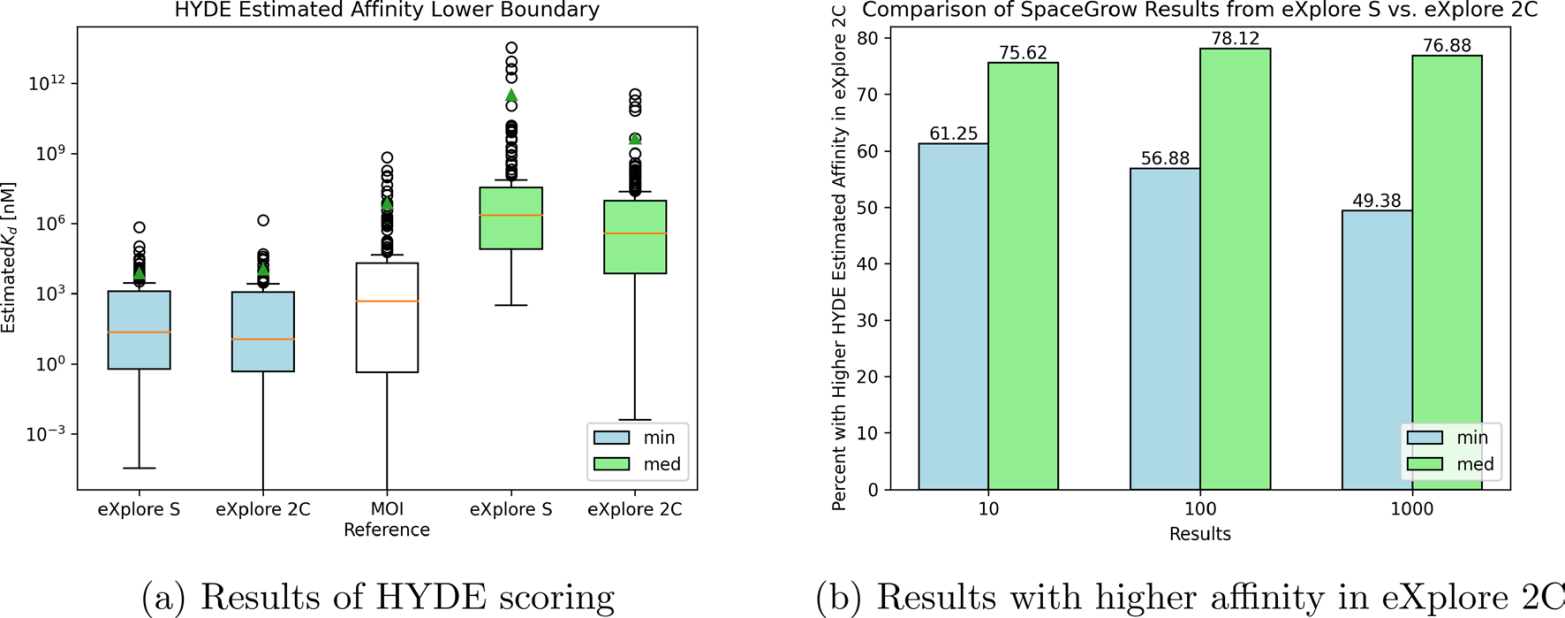
Estimated affinity of SpaceGrow results when evaluated with HYDE on the PDBbind protein structures associated with the respective 160 MOIs. Results were evaluated (**a**) by HYDE score given as the lower boundary of the estimated affinity. Minimum HYDE scores (min) for the top thousand SpaceGrow results are shown as blue boxes. Median HYDE scores (med) for the top thousand SpaceGrow results are shown as green boxes. The orange line indicates the median value of the 160 data points. The green triangle marks the average value of the 160 data points. Estimated affinity lower boundary for all 160 MOIs in their native binding mode is depicted as a reference in the middle. In (**b**) results were evaluated by the percentage of MOIs for which results from the larger eXplore 2C space had a lower estimated $K_d$ value, i.e. a higher affinity. The min value only takes the best HYDE score into consideration while the median compares the median HYDE score of the top thousand results for each search

To further analyze the improvement in screening the larger space and to assess the SpaceGrow results using an independent orthogonal strategy, HYDE was used to analyze the ligand candidates for their binding affinity at the interaction site. The results are summarized in Fig. 7. HYDE provides an upper and a lower bound for the estimated affinity that correlate with each other. For reasons of convenience, only the lower bound was used for our evaluations.

The estimated affinity is shown as a lower boundary of the $K_d$ value for the MOIs and the ligand candidates, so lower values mean a higher affinity of the molecule. The best as well as the median HYDE scores for the 160 MOIs are depicted in Fig. 7a with a logarithmic scale. This should also be noted when comparing the HYDE scores generated for results from the eXplore S and the eXplore 2C. The distribution of the best scores is comparable for the results from both spaces. However, the median of the scores implies that potentially better binders can be mined from the larger space.

For visual comparison of the results’ HYDE scores, percentages of ligands retrieved from the larger space displaying improved calculated affinity compared to their MOI counterpart are depicted in Fig. 7b. When considering for each MOI only the SpaceGrow result with the best HYDE score, this percentage decreases from about 60 % within the top ten to about 50 % within the top thousand results. Considering the median HYDE score of the result enables a comparison of the results from the different spaces beyond first rank, again showing a benefit when using the larger space. For the top ten, hundred and thousand results, in over 75 % of the cases a better median expected affinity was achieved when using the larger space.

**Fig. 8:**
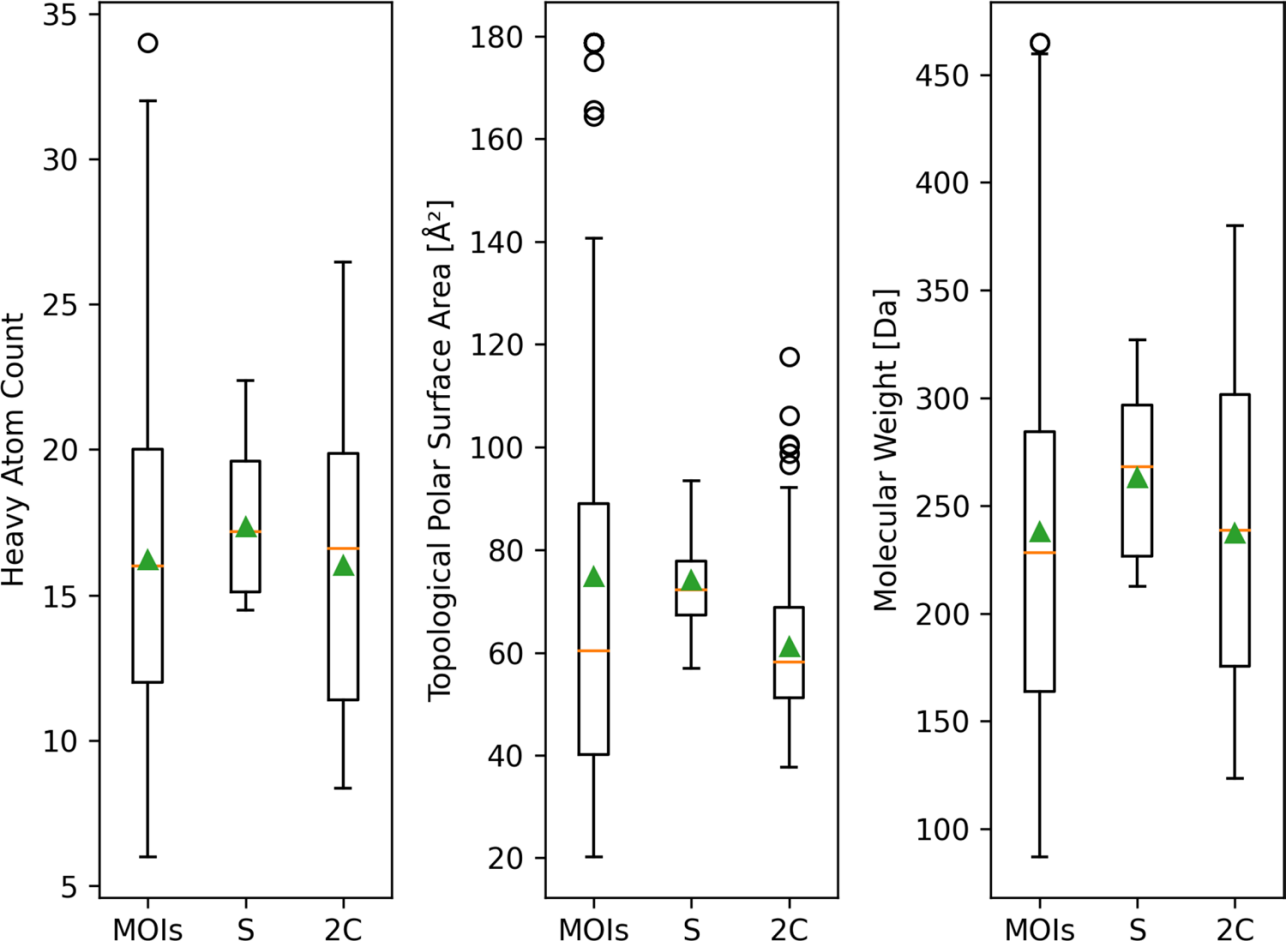
Averaged molecular properties of the top thousand result molecules for each search of the 160 MOIs in the eXplore S (S) and the eXplore 2C (2C). The properties of the MOIs are depicted as well. The orange line indicates the median value. The green triangle marks the average value

When it comes to the diversity of the result molecules, Fig. 8 shows statistics on the average heavy atom count, topological polar surface area and molecular weight of the top thousand result molecules. The properties of the MOIs are shown as well. The MOIs are diverse in their size, containing between six and 34 heavy atoms and show a molecular weight between 87 and 465 Da. Overall, the MOIs had an average number of heavy atoms of 16 and an average molecular weight of 238 Da. Comparing the results found in eXplore S and eXplore 2C, the results from eXplore 2C are more spread for all analyzed properties. Also, the median and average of the values are smaller for all properties when searching in the larger space. However, Figure 6 of the Supplementary Information reveals that regarding results of a single search for an MOI, the ranges of each property are smaller for results from eXplore 2C compared to results from eXplore S. The heavy atom count and molecular weight ranges of molecules found within the larger space tend to be closer to the corresponding values of the MOI, which was used as the query.

**Fig. 9:**
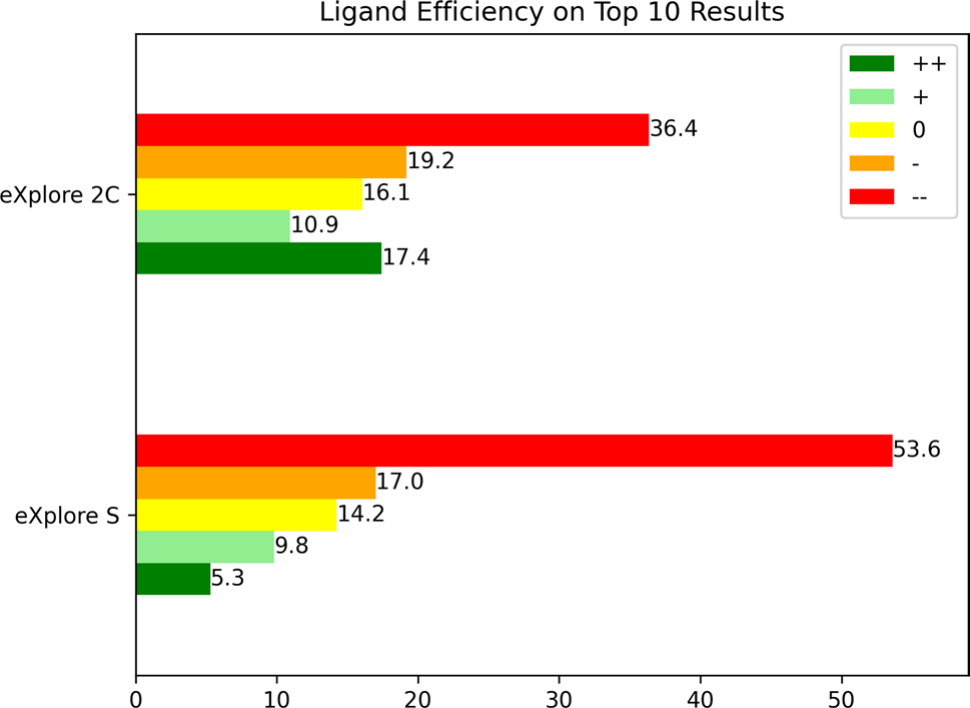
Ligand efficiency classes predicted by HYDE for the top ten result molecules for each search of the 160 MOIs in the eXplore S and the eXplore 2C. Bars provide the percentage of molecules which were rated according to the underlying ligand efficiency class. Dark green ($++$) is rated as best and red ($--$) as least efficient

Besides estimated affinity, HYDE also classifies molecules according to their ligand efficiency into five categories, i.e. from best to least efficient ($++, +, 0, -, --$, respectively). The ligand efficiencies for the top 10 ranking results of each MOI query are summarized in Fig. 9. Regarding the results of the smaller space, 53.6 % of the results were classified into the least efficient category while only 5.3 % were classified into the best category. Searching in the large space, molecules in the last category were reduced to 36.5 % and 17.4 % were classified into the best category. This trend is can also be observed among the top hundred and top thousand results, as the corresponding Figure 8 in the Supplementary Information confirms.

The differences between the top results per search found in the differently sized spaces become evident considering the median scores. The median SpaceGrow score increased when searching in the larger space, implying results with a higher molecular shape similarity between the results and the MOIs. Analyzing the HYDE scores on the top ten, hundred, and thousand results shows that for over 75 % a higher median affinity was predicted for results of the larger space. However, a closer look on the results revealed that there was only one result found in both spaces when comparing the top thousand results for each MOI found on both spaces. The larger space provides more synthons and thus also provides more high scoring results. Still, the evaluations of the average heavy atom count, topological polar surface area and molecular weight show wider ranges of the values for the results of the larger space. This indicates a more diverse selection of molecules with the opportunity of more available synthons. Another observation was the lower average and median of these properties in the results from searching in the larger space. As the larger space contains more synthons and SpaceGrow penalizes aligned molecules which are larger than the MOI, molecules with a better shape fit are smaller in general. Since size in terms of the number of heavy atoms also has an influence on ligand efficiency, the smaller results of the larger space are categorized into better efficiency classes. Thus, results from the larger space are likely to contain a higher number of molecules with similar binding capabilities as the query.

### Run time evaluations

To search in chemical spaces with SpaceGrow, a space has to be converted into a database with a suitable format and 3D conformer ensembles for the synthons have to be generated. On an eight core machine with an 11th Gen Intel(R) Core(TM) i9 processor (2.5 Ghz) with 32 GB memory and Windows 11, the generation of the database with the synthons of eXplore 2C took approx. 4.5 h. The generation of the database for eXplore S took less than 4 min.

While performing the searches with the 160 selected MOIs for the above experiment, the run time of SpaceGrow was evaluated for both spaces. Each search ran using one thread (on a single CPU core) of a machine in a heterogenous linux compute cluster. The SpaceGrow process can be divided into three parts. First, in a preprocessing step, the database is checked to be valid and loaded. Next, the screening includes the partitioning of the MOI and the generation of the corresponding descriptors, the comparison of the descriptors to those of the database, and picking the best scoring molecules as results. Finally, the molecule construction combines the synthons according to the underlying reaction rules.

**Fig. 10:**
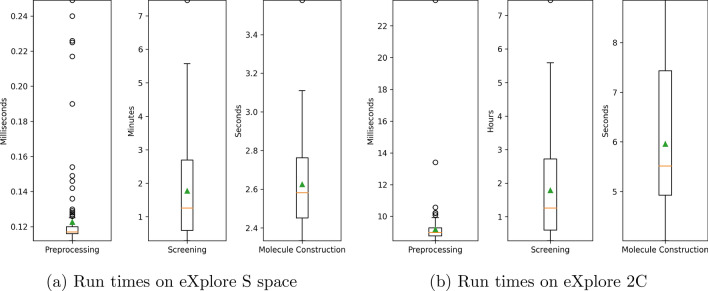
SpaceGrow run times (wall clock time) of preprocessing, screening and result construction process. The 160 MOIs were used to search (**a**) in the eXplore S and (**b**) in the eXplore 2C. Calculations were performed on a single CPU core of a heterogenous compute cluster. Note that every boxplot has its own scale. The median is depicted as an orange line and the average as a green triangle

In Fig. 10 the three parts are depicted separately. On both spaces, preprocessing is a matter of milliseconds and molecule construction is done in less than ten seconds. While the median screening time on the small space is less than 1.5 min, on the large space it is less than 1.5 h. The average screening time is less than 2 min or 2 h, respectively. The run time of the screening scales with the number of descriptor comparisons. Thus the number of synthons in the space has a huge impact. While the small space is composed of approx. 10,000 synthons, the large space consists of approx. 400,000 synthons.

**Fig. 11:**
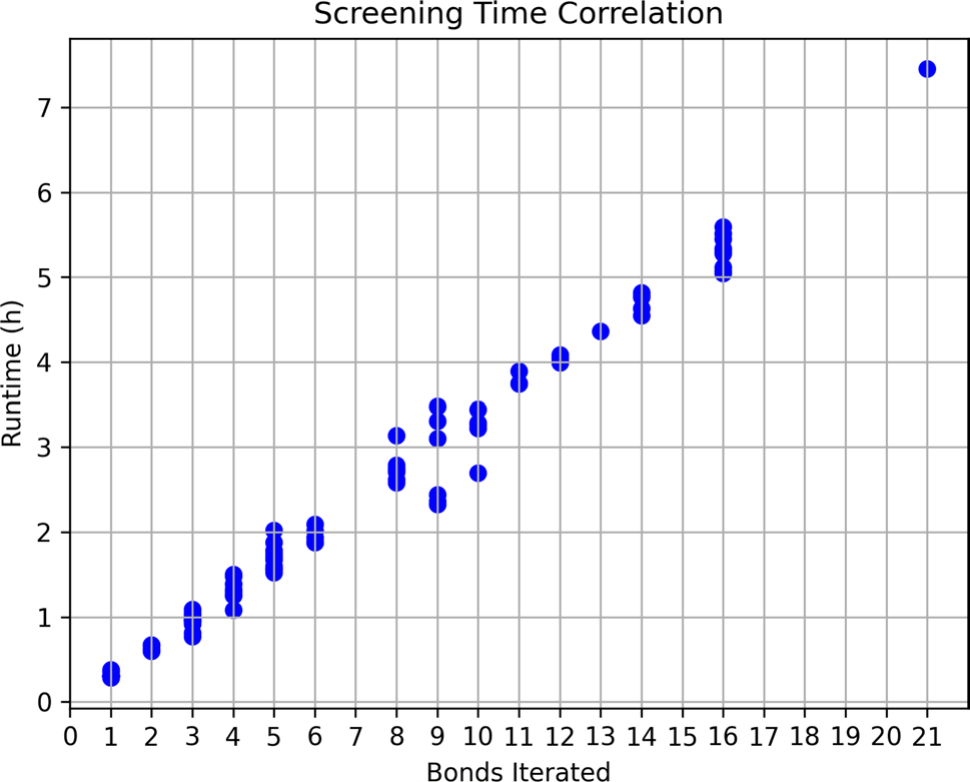
Correlation of number of bonds in the MOI iterated and run time of the search in the eXplore 2C

The shortest run time on the large space was about half an hour and the longest run time over seven h. Next to the number of synthons, the number of acyclic bonds within the MOI is an important factor for the run time. As Fig. 11 shows the run time correlates linearly with the number of acyclic bonds in the MOI, i.e. the number of iterations in SpaceGrow. In each iteration, a pose scoring of the entire database is performed and a descriptor for both parts of the partitioned MOI is performed. Overall, using SpaceGrow, the large eXplore 2C can be searched within h on a single CPU of a regular desktop computer.

### Application: mining for potential GPCR binders

 To elucidate the relevance of SpaceGrow for drug discovery purposes, we performed an analysis on four members of the GPCR family with the aim to discover chemically novel potential binders.

**Fig. 12:**
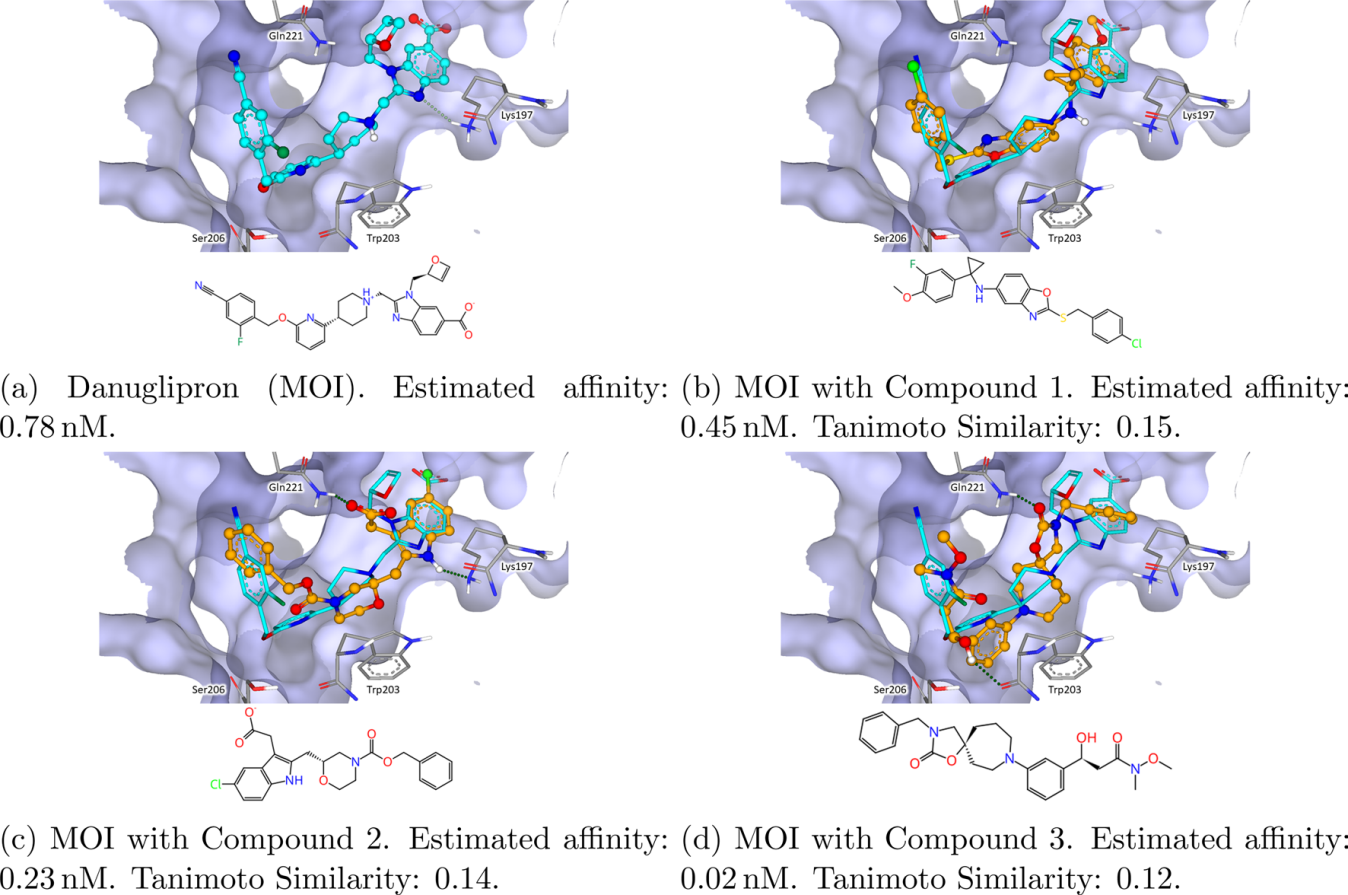
Structure of the human glucagon-like peptide-1 receptor (PDB-ID: 7LCK) with danuglipron as MOI and HYDE optimized SpaceGrow results. The MOI is colored light blue. SpaceGrow results are colored orange. Estimated affinity is provided as the reported lower bound of the HYDE score

Our first GPCR of interest is the human glucagon-like peptide-1 receptor (GLP-1R). It is captured with danuglipron (PF 06882961, PDB-ID: 7LCK). [65, 66] Danuglipron is of current interest, since it is under investigation in clinical trial for the treatment of type 2 diabetes and obesity. [67–73] Fig. 12 shows Danuglipron within the binding site of GLP-1R (a) and the overlay with three retrieved results (b-d), together with the estimated affinity and the respective Tanimoto similarity calculated using the Morgan Fingerprint implementation with radius four from RDKit. [64]. Compound 1 shows a good superposition due to sharing several structural features of danuglipron. This includes the positioning of the chlorine atom on the nitrile group of the query. The middle of the molecule shows a replacement of the central ring system with a benzoisoxazole which can be considered a scaffold hop. This is also reflected by the low Tanimoto similarity. Overall, the results found for GLP-1R provided different scaffolds and various interaction patterns. By picking compounds from eXplore 2C, it is also likely that the found compounds are easy to synthesize. The other top ten SpaceGrow results after filtering for this MOI and all following can be found in Table 1 of the Supplementary Information.

**Fig. 13:**
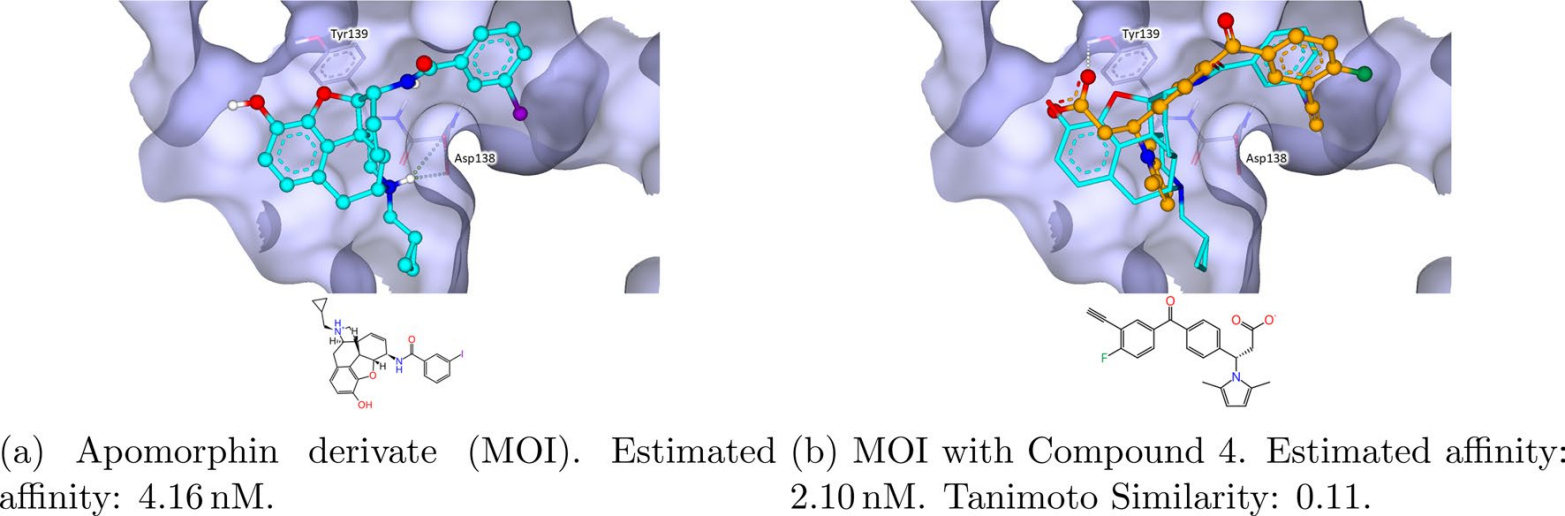
Structure of the $\kappa$-opioid receptor (PDB-ID: 6B73) with an apomorphin derivate as MOI and HYDE optimized SpaceGrow results. The MOI is colored light blue. SpaceGrow results are colored orange. Estimated affinity is provided as the reported lower bound of the HYDE score

The next GPCR investigated was the $\kappa$-opioid receptor ($\kappa$-OR), which is responsible for hallucinogenic, dysphoric, and analgesic activities induced by opioids. With the PDB-ID 6B73 [74, 75] of the nanobody-stabilized active state, an apomorphin derivate was co-crystalized. This ligand served as an MOI for a SpaceGrow search. The result shown in Fig. 13 has an alkynyl group which can be considered as a bioisostere to the iodine group of the MOI. Overall, the results show a series of bioisosteres, which are more chemically accessible than the MOI which belongs to the class of natural products. All ten analyzed result compounds contain an imidazole with two methyl groups predicted to interact as dowels that improve the fit of the ligand within the pocket.

**Fig. 14:**
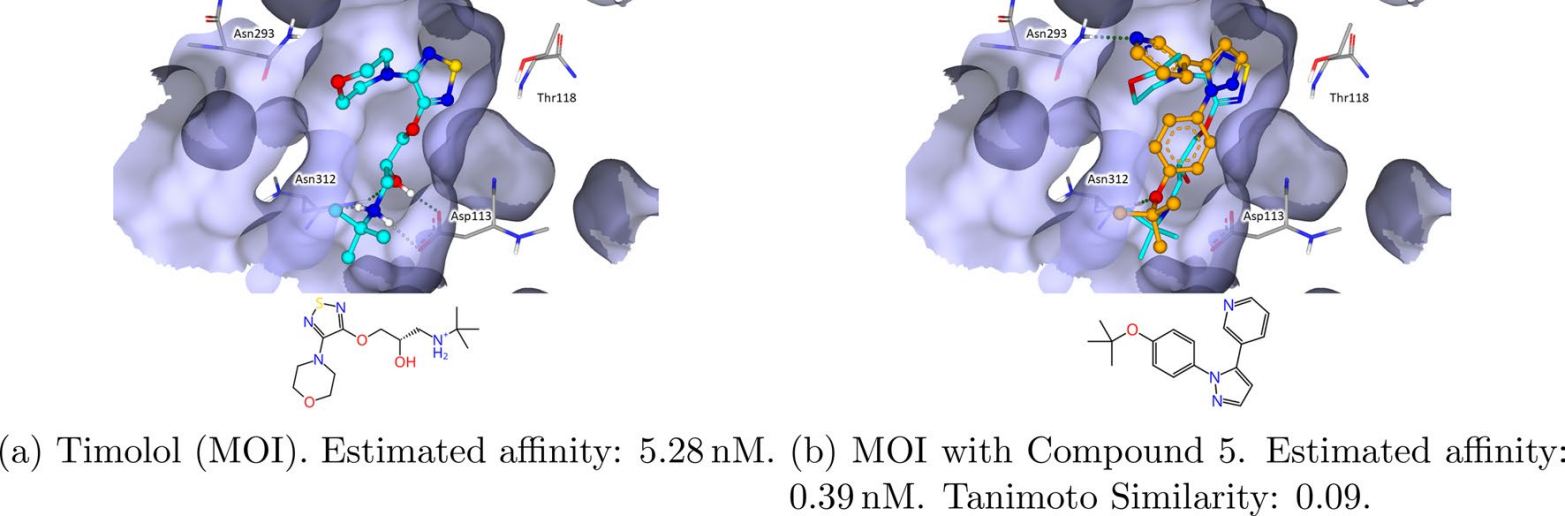
Structure of the $\beta$-2 adrenergic receptor (PDB-ID: 6PS1) with timolol as MOI and HYDE optimized SpaceGrow results. The MOI is colored light blue. SpaceGrow results are colored orange. Estimated affinity is provided as the reported lower bound of the HYDE score

$\beta$-2 adrenergic receptors (ADRB2) are targets for asthma [76] and COPD [77]. Also, ADRB2 regulates the phenotype and metabolism of adipose and smooth skeletal muscle tissue and had therefore recently been discussed as a re-emerging target to combat obesity. [78] It has also been discussed in context of neural disease development [79] and cardiac fibroblast autophagy. [80] Timolol was cocrystalized with ADRB2 within the structure 6PS1 [55, 81]. Back in 1997, Timolol was already introduced as a beta-adrenergic blocking agent to treat glaucoma. [82] Using Timolol as an MOI, a result of SpaceGrow is shown in Fig. 14. Compound 5 shows a good shape overlay matching the isopropyl group and the ring systems of five atoms. In the superposition, H-bond interactions between the pyridine moiety with Asn293 and the ether group with Asn312 are feasible. Even though most active compounds acting at ADRB2 contain a protonated amine, a class of ligands lacking a charge at physiological pH 7.4 has been reported [83]. The series of the ten considered results in general shows many 3D derivates with a high similarity to Compound 5 but various decorations. Also, the series contains the ether group that could form possible interactions.

**Fig. 15:**
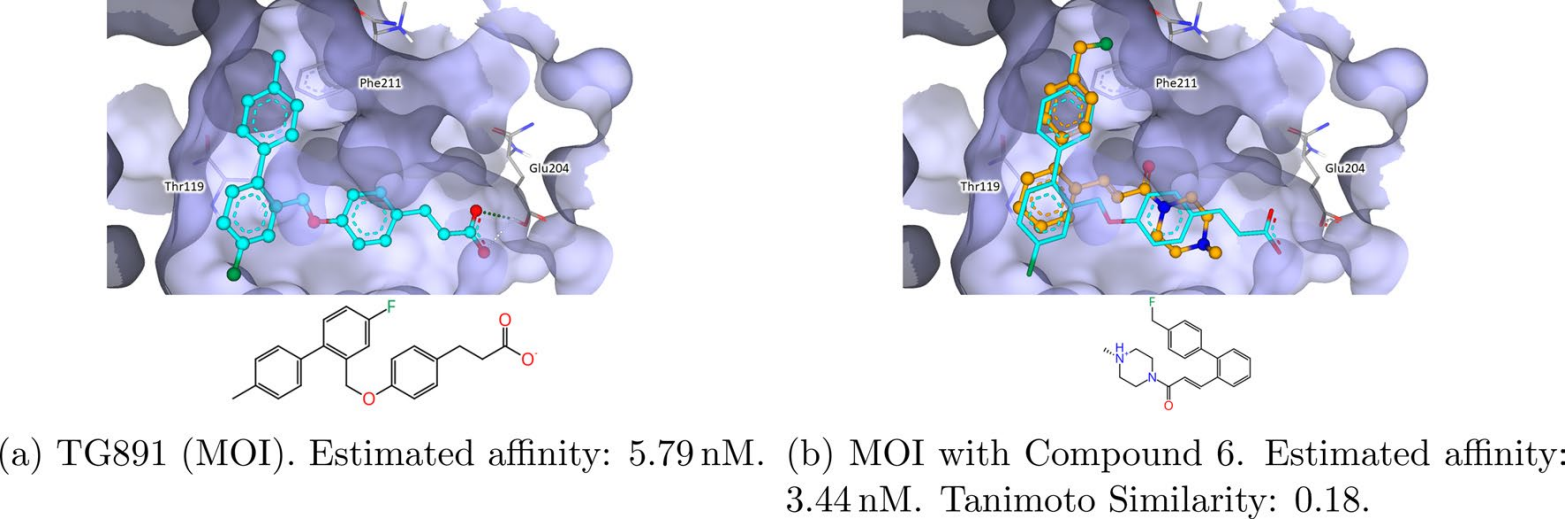
Structure of the Free Fatty acid receptor GPR120 (PDB-ID: 8ID8) with TG891 as MOI and HYDE optimized SpaceGrow results. The MOI is colored light blue. SpaceGrow results are colored orange. Estimated affinity is provided as the reported lower bound of the HYDE score

The Free Fatty acid receptor 4 (FFAR4/GPR120) mediates potent anti-inflammatory and insulin sensitizing effects. [84]. The structure with the PDB-ID 8ID8 captures GPR120 with the selective GPR120 agonist TUG891 [85, 86]. Figure 15 shows the results of using TG891 as the MOI to screen the eXplore 2C with SpaceGrow. Compound 6 shows a good shape match and overall matches the pocket well. The fluorine group complements the lipophilic properties of the pocket. The protonated piperazine allows for potential interaction with carboxylic acids. The ligand structure contains a Michael acceptor in close proximity to Thr119 and which may increase the duration stay of the ligand at the receptor. Overall, the results for the MOI TUG891 can be split into two series. One series contains the Michael acceptor, whereas the other replaced it by a simple amide, as can be seen in Table 4 of the Supplementary Information.

## Conclusions

SpaceGrow is a solely shape-based approach for screening ultra-large chemical spaces. For validation-purposes a sample space was created and also fully enumerated for the comparison to a freely available state of the art superposition tool. The sample space consists of the fragments of a set of molecules of interest as well as their analogs. The corresponding fully enumerated library consists of all valid pairs of coupled fragments. SpaceGrow was able to retrieve on top ranks a significantly higher number of both, the molecules of interest and their analogs, compared to a conventional method from the enumerated set. When comparing RMSD values of the reproduced poses of the MOIs and the analogs, SpaceGrow shows to produce very reasonable alignments. Searching in a comparatively small ($10^4$) and a much larger ($10^9$) subset of a tailored version of the trillion-sized eXplore space with SpaceGrow gave a glimpse on the advantages of being able to search in larger and larger spaces of molecules. The median SpaceGrow score of the top thousand results per search was found to be over thirty times higher using the larger space, indicating a significantly higher shape similarity. In order to check for the relevance of these search results, we also used the generated poses to calculate estimated binding affinities for the given target structure. The median estimated affinity of the top ten, top hundred, and top thousand results, was higher in over 75 % of the results from the larger space. The average heavy atom count, however, was smaller for the results from the larger space leading to overall more efficient ligands with a higher probability to be a viable substitute for the MOI used for searching. The run time of SpaceGrow scales roughly linearly with the number of acyclic bonds in the molecule of interest as well as the number of fragments spanning the space. This is the major advantage over conventional search methods that scale with the number of product molecules. This way of avoiding the combinatorial explosion is of key importance for the ability to screen combinatorial chemical spaces worth billions and even trillions of molecules. The benefits of mining chemical spaces in the drug discovery process, using 3D virtual screening methods has already been proven by other applications. [31–34] Applying the SpaceGrow workflow on a number of GPCR targets allowed for a closer look on the quality of results and the potential of this method. The compounds retrieved, showed a potential for scaffold hopping, generally a high estimated affinity and a low Tanimoto similarity compared to the MOI. Therefore, SpaceGrow is able to retrieve compounds from ultra-large spaces that offer novel chemotypes to explore. The current implementation of SpaceGrow should be considered a proof of concept that shows the benefits of 3D shape-based screening in large chemical spaces. A future goal must be an extension of this concept that allows to work with three and four-component reactions as well. This would enable searching in even larger chemical spaces taking full advantage of the currently available make-on-demand libraries from a number of compound vendors. Another future research topic is an extension of the SpaceGrow score, which is currently shape-based only. Incorporating interactions could further improve the matching of polar atoms when comparing the SpaceGrow descriptors of molecules early in the workflow. To increase the diversity and molecular novelty of the resulting molecules, a clustering mechanism could be included in addition to the SpaceGrow score.

## Supplementary Information

Below is the link to the electronic supplementary material.

Supplementary file 1 (pdf 33490 KB)

## Data Availability

SpaceGrow, FastGrowDBCreator and the tailored eXplore 2C space are available on demand by BioSolveIT GmbH. Supplementary Material is provided for download from the datasets web page of Universität Hamburg, ZBH - Center for Bioinformatics (download).

## References

[1] van Hilten N, Chevillard F, Kolb P (2019) Virtual compound libraries in computer-assisted drug discovery. J Chem Inform Modeling 59:644–651 10.1021/acs.jcim.8b00737 30624918

[2] Walters WP (2018) Virtual chemical libraries: miniperspective. J Med Chem 62:1116–1124 30148631 10.1021/acs.jmedchem.8b01048

[3] Chevillard F, Kolb P (2015) SCUBIDOO: a large yet screenable and easily searchable database of computationally created chemical compounds optimized toward high likelihood of synthetic tractability. J Chem Inform Modeling 55:1824–1835 10.1021/acs.jcim.5b00203 26282054

[4] Humbeck L, Weigang S, Schäfer T, Mutzel P, Koch O (2018) CHIPMUNK: a virtual synthesizable small-molecule library for medicinal chemistry, exploitable protein-protein interaction modulators. ChemMedChem 13:532–539 29392860 10.1002/cmdc.201700689

[5] Korn M, Ehrt C, Ruggiu F, Gastreich M, Rarey M (2023) Navigating large chemical spaces in early-phase drug discovery. Curr Opin Struct Biol 80:102578 37019067 10.1016/j.sbi.2023.102578

[6] Warr WA, Nicklaus MC, Nicolaou CA, Rarey M (2022) Exploration of ultralarge compound collections for drug discovery. J Chem Inform Modeling 62:2021–2034 10.1021/acs.jcim.2c00224 35421301

[7] Hoffmann T, Gastreich M (2019) The next level in chemical space navigation: going far beyond enumerable compound libraries. Drug Discov Today 24:1148–1156 30851414 10.1016/j.drudis.2019.02.013

[8] Klingler F-M, Gastreich M, Grygorenko OO, Savych O, Borysko P, Griniukova A, Gubina KE, Lemmen C, Moroz YS (2019) SAR by space: enriching hit sets from the chemical space. Molecules 24:3096 31454992 10.3390/molecules24173096 PMC6749418

[9] Chevillard F, Rimmer H, Betti C, Pardon E, Ballet S, van Hilten N, Steyaert J, Diederich WE, Kolb P (2018) Binding-site compatible fragment growing applied to the design of 2-adrenergic receptor ligands. J Med Chem 61:1118–1129 29364664 10.1021/acs.jmedchem.7b01558

[10] Virshup AM, Contreras-García J, Wipf P, Yang W, Beratan DN (2013) Stochastic voyages into uncharted chemical space produce a representative library of all possible drug-like compounds. J Am Chem Soc 135:7296–7303 23548177 10.1021/ja401184g PMC3670418

[11] van Deursen R, Reymond J-L (2007) Chemical space travel. ChemMedChem: Chem Enabling Drug Discov 2:636–640 10.1002/cmdc.200700021 17366512

[12] Detering C, Claussen H, Gastreich M, Lemmen C (2010) KnowledgeSpace-a publicly available virtual chemistry space. J Cheminform 2:1–120298528

[13] Hu Q, Peng Z, Kostrowicki J, Kuki A (2011) LEAP into the Pfizer Global Virtual Library (PGVL) space: creation of readily synthesizable design ideas automatically. Chem Lib Design. 10.1007/978-1-60761-931-4_13 10.1007/978-1-60761-931-4_13 20981528

[14] Nicolaou CA, Watson IA, Hu H, Wang J (2016) The proximal lilly collection: mapping, exploring and exploiting feasible chemical space. J Chem Inform Modeling 56:1253–1266 10.1021/acs.jcim.6b00173 27286472

[15] Boehm M, Wu T-Y, Claussen H, Lemmen C (2008) Similarity searching and scaffold hopping in synthetically accessible combinatorial chemistry spaces. J Med Chem 51:2468–2480 18380426 10.1021/jm0707727

[16] Lessel U, Wellenzohn B, Lilienthal M, Claussen H (2009) Searching fragment spaces with feature trees. J Chem Inform Modeling 49:270–279 10.1021/ci800272a 19434829

[17] Warr W. Report on an NIH workshop on ultralarge chemistry databases. **2021**,

[18] (2023)Enamine, Enamine REAL Space. https://enamine.net/compound-collections/real-compounds/real-space-navigator, accessed on November 01

[19] WuXi LabNetwork, GalaXi Space. https://www.labnetwork.com/frontend-app/p/#!/library/virtual, accessed on November 01, (2023)

[20] OTAVAchemicals, CHEMriya Space. https://www.otavachemicals.com/products/chemriya, accessed on November 01, (2023)

[21] Neumann A, Marrison L, Klein R (2023) Relevance of the trillion-sized chemical space “eXplore’’ as a source for drug discovery. ACS Med Chem Lett 14:466–472 37077402 10.1021/acsmedchemlett.3c00021 PMC10108389

[22] Chemspace, Freedom Space. https://chem-space.com/compounds/freedom-space, accessed on November 01, (2023)

[23] Pottel J, Moitessier N (2017) Customizable generation of synthetically accessible, local chemical subspaces. J Chem Inform Modeling 57:454–467 10.1021/acs.jcim.6b00648 28234470

[24] Zabolotna Y, Volochnyuk DM, Ryabukhin SV, Gavrylenko K, Horvath D, Klimchuk O, Oksiuta O, Marcou G, Varnek A (2021) SynthI: a new open-source tool for synthon-based library design. J Chem Inform Modeling 62:2151–2163 10.1021/acs.jcim.1c00754 34723532

[25] Wahl J, Sander T (2022) Fully automated creation of virtual chemical fragment spaces using the open-source library OpenChemLib. J Chem Inform Modeling 62:2202–2211 10.1021/acs.jcim.1c01041 35073086

[26] Fischer JR, Lessel U, Rarey M (2011) Improving similarity-driven library design: customized matching and regioselective feature trees. J Chem Inform Modeling 51:2156–2163 10.1021/ci200014g 21848342

[27] Brown N (2013). Feature Trees. 10.1002/9783527665143.ch09

[28] Bellmann L, Penner P, Rarey M (2020) Topological similarity search in large combinatorial fragment spaces. J Chem Inform Modeling 61:238–251 10.1021/acs.jcim.0c00850 33084338

[29] Schmidt R, Klein R, Rarey M (2021) Maximum common substructure searching in combinatorial make-on-demand compound spaces. J Chem Inform Modeling 62:2133–2150 10.1021/acs.jcim.1c00640 34478299

[30] Degen J, Rarey M (2006) FlexNovo: structure-based searching in large fragment spaces. ChemMedChem: Chem Enabling Drug Discov 1:854–868 10.1002/cmdc.200500102 16902939

[31] Sadybekov AA et al (2022) Synthon-based ligand discovery in virtual libraries of over 11 billion compounds. Nature 601:452–459 34912117 10.1038/s41586-021-04220-9 PMC9763054

[32] Muller J et al (2022) Magnet for the needle in haystack: crystal structure first Fragment hits unlock active chemical matter using targeted exploration of vast chemical spaces. J Med Chem 65:15663–15678 36069712 10.1021/acs.jmedchem.2c00813

[33] Beroza P, Crawford JJ, Ganichkin O, Gendelev L, Harris SF, Klein R, Miu A, Steinbacher S, Klingler F-M, Lemmen C (2022) Chemical space docking enables large-scale structure-based virtual screening to discover ROCK1 kinase inhibitors. Nat Commun 13:6447 36307407 10.1038/s41467-022-33981-8 PMC9616902

[34] Meyenburg C, Dolfus U, Briem H, Rarey M (2023) Galileo: three-dimensional searching in large combinatorial fragment spaces on the example of pharmacophores. J Comput-Aided Mole Design 37:1–16 10.1007/s10822-022-00485-y PMC10032335 36418668

[35] Hönig SM, Lemmen C, Rarey M (2023) Small molecule superposition: a comprehensive overview on pose scoring of the latest methods. Wiley Interdiscip Rev: Comput Mole Sci 13:e1640

[36] Grant JA, Gallardo MA, Pickup BT (1996) A fast method of molecular shape comparison: a simple application of a Gaussian description of molecular shape. J Comput Chem 17:1653–1666

[37] Open Eye Scientific Software, Santa Fe, NM, ROCS. 2006; https://www.eyesopen.com/rocs, accessed on November 28, (2023)

[38] Lemmen C, Lengauer T, Klebe G (1998) FlexS: a method for fast flexible ligand superposition. J Med Chem 41:4502–4520 9804690 10.1021/jm981037l

[39] Lemmen C, Lengauer T (1997) Time-efficient flexible superposition of medium-sized molecules. J Comput-Aided Mole Design 11:357–368 10.1023/a:1007959729800 9334902

[40] FlexS Version 5.0.0, BioSolveIT GmbH, St. Augustin, Germany, (2023), biosolveit.de/FlexS

[41] Chan SL, Labute P (2010) Training a scoring function for the alignment of small molecules. J Chem Inform modeling 50:1724–1735 10.1021/ci100227h PMC2946173 20831240

[42] Molecular Operating Environment (MOE). https://www.chemcomp.com/Products.htm, accessed on November 28, 2023

[43] FastROCS Toolkit | Real-Time Shape Similarity | Lead Discovery. https://www.eyesopen.com/molecular-modeling-fastrocs, accessed on November 28, 2023

[44] Penner P, Martiny V, Gohier A, Gastreich M, Ducrot P, Brown D, Rarey M (2020) Shape-based descriptors for efficient structure-based fragment growing. J Chem Inform Modeling 60:6269–6281 10.1021/acs.jcim.0c00920 33196169

[45] Penner P, Martiny V, Bellmann L, Flachsenberg F, Gastreich M, Theret I, Meyer C, Rarey M (2022) FastGrow: on-the-fly growing and its application to DYRK1A. J Comput -Aided Mole Design 36:639–651 10.1007/s10822-022-00469-y PMC9512872 35989379

[46] Wang R, Fang X, Lu Y, Wang S (2004) The PDBbind database: collection of binding affinities for protein- ligand complexes with known three-dimensional structures. J Med Chem 47:2977–2980 15163179 10.1021/jm030580l

[47] Wang R, Fang X, Lu Y, Yang C-Y, Wang S (2005) The PDBbind database: methodologies and updates. J Med Chem 48:4111–4119 15943484 10.1021/jm048957q

[48] Hu J, Liu Z, Yu D-J, Zhang Y (2018) LS-align: an atom-level, flexible ligand structural alignment algorithm for high-throughput virtual screening. Bioinformatics 34:2209–2218 29462237 10.1093/bioinformatics/bty081 PMC6022693

[49] Schneider N, Lange G, Hindle S, Klein R, Rarey M (2013) A consistent description of HYdrogen bond and DEhydration energies in protein-ligand complexes: methods behind the HYDE scoring function. J Comput-Aided Mole Design 27:15–29 10.1007/s10822-012-9626-2 23269578

[50] Reulecke I, Lange G, Albrecht J, Klein R, Rarey M (2008) Towards an integrated description of hydrogen bonding and dehydration: decreasing false positives in virtual screening with the HYDE scoring function. ChemMedChem: Chem Enabling Drug Discov 3:885–897 10.1002/cmdc.200700319 18384086

[51] CoLibri Version 8.0, BioSolveIT GmbH, St. Augustin, Germany, (2023), biosolveit.de/CoLibri

[52] Jung S, Klein R, Gastreich M (2024) CoLibri Commandline Documentation 8.0. https://www.biosolveit.de/wp-content/uploads/2023/03/CoLibri-UserGuide.pdf, accessed on January 22,

[53] FastGrow Version 1.1.0, BioSolveIT GmbH, St. Augustin, Germany, (2023), biosolveit.de/FastGrow

[54] Jung S, Gastreich M, Flachsenberg F (2024) FastGrow Commandline Documentation Version 1.1. https://www.biosolveit.de/wp-content/uploads/2023/05/FastGrow-UserGuide.pdf, accessed on January 22,

[55] Liu Z et al (2019) Discovery of potent inhibitors of 11-hydroxysteroid dehydrogenase type 1 using a novel growth-based protocol of in silico screening and optimization in CONTOUR. J Chem Inform Modeling 59:3422–3436 10.1021/acs.jcim.9b00198 31355641

[56] Malhotra S, Karanicolas J (2017) When does chemical elaboration induce a ligand to change its binding mode? J Med Chem 60:128–145 27982595 10.1021/acs.jmedchem.6b00725 PMC5525026

[57] Bietz S, Rarey M (2016) SIENA: efficient compilation of selective protein binding site ensembles. J Chem Inform Modeling 56:248–259 10.1021/acs.jcim.5b00588 26759067

[58] eMolecules, eXplore the world’s largest commercially available chemical space. https://www.emolecules.com/explore, accessed on December 17, (2023)

[59] BioSolveIT GmbH, eXplore Cookbook. https://www.biosolveit.de/infiniSee/cookbook, accessed on January 22, (2024)

[60] HYDE Version 2.0.0, BioSolveIT GmbH, St. Augustin, Germany, 2023, biosolveit.de/HYDE

[61] Sriram K, Insel PA (2018) G protein-coupled receptors as targets for approved drugs: how many targets and how many drugs? Mole Pharmacol 93:251–258 10.1124/mol.117.111062 PMC5820538 29298813

[62] Munk C, Mutt E, Isberg V, Nikolajsen LF, Bibbe JM, Flock T, Hanson MA, Stevens RC, Deupi X, Gloriam DE (2019) An online resource for GPCR structure determination and analysis. Nat Methods 16:151–162 30664776 10.1038/s41592-018-0302-x PMC6881186

[63] Pándy-Szekeres G, Caroli J, Mamyrbekov A, Kermani AA, Keserű GM, Kooistra AJ, Gloriam DE (2023) GPCRdb in 2023: state-specific structure models using AlphaFold2 and new ligand resources. Nucleic Acids Res 51:D395–D402 36395823 10.1093/nar/gkac1013 PMC9825476

[64] Landrum G (2023). RDKit https://www.rdkit.org/, accessed on November 03

[65] Belousoff M, Johnson R, Drulyte I, Yu L, Kotecha RA, Danev Wootten D, Zhang X, Sexton P (2023) RCSB PDB - PF 06882961 bound to the glucagon-like peptide-1 receptor (GLP-1R). 10.2210/pdb7LCK/pdb, accessed on December 08,

[66] Zhang X, Johnson RM, Drulyte I, Yu L, Kotecha A, Danev R, Wootten D, Sexton PM, Belousoff MJ (2021) Evolving cryo-EM structural approaches for GPCR drug discovery. Structure 29:963–974 33957078 10.1016/j.str.2021.04.008

[67] Saxena AR, Gorman DN, Esquejo RM, Bergman A, Chidsey K, Buckeridge C, Griffith DA, Kim AM (2021) Danuglipron (PF-06882961) in type 2 diabetes: a randomized, placebo-controlled, multiple ascending-dose phase 1 trial. Nat Med 27:1079–1087 34127852 10.1038/s41591-021-01391-w

[68] Ono R, Furihata K, Ichikawa Y, Nakazuru Y, Bergman A, Gorman DN, Saxena AR (2023) A phase 1 study to evaluate the safety, tolerability, pharmacokinetics and pharmacodynamics of danuglipron (PF-06882961), an oral small-molecule glucagon-like peptide-1 receptor agonist, in Japanese adults with type 2 diabetes mellitus. Diabetes Obes Metab 25:805–814 36433713 10.1111/dom.14928 PMC10107991

[69] Fediuk DJ, Gorman DN, Stoddard S-A, Zhang Y, Ogden AG, Winton JA, Saxena AR (2023) Effect of renal impairment on the pharmacokinetics of a single oral dose of danuglipron in participants with type 2 diabetes. J Clin Pharmacol. 10.1002/jcph.2371 37840155 10.1002/jcph.2371

[70] Saxena AR, Frias JP, Brown LS, Gorman DN, Vasas S, Tsamandouras N, Birnbaum MJ (2023) Efficacy and safety of Oral small molecule glucagon-like peptide 1 receptor agonist Danuglipron for glycemic control among patients with type 2 diabetes: a randomized clinical trial. JAMA Network Open 6:e2314493–e2314493 37213102 10.1001/jamanetworkopen.2023.14493 PMC10203889

[71] Fatima H, Rangwala HS, Mustafa MS, Shafique MA, Abbas SR, Rizwan A, Fadlalla Ahmed TK, Arshad A (2023) Evaluating glycemic control efficacy and safety of the oral small molecule glucagon-like peptide 1 receptor agonist danuglipron in type 2 diabetes patients: a systemic review and meta-analysis. Diabetes Metab Syndr Obes 16:3567–3578 37954886 10.2147/DMSO.S439587 PMC10638946

[72] Saxena AR, Frias JP, Gorman DN, Lopez RN, Andrawis N, Tsamandouras N, Birnbaum MJ (2023) Tolerability, safety and pharmacodynamics of oral, small-molecule glucagon-like peptide-1 receptor agonist danuglipron for type 2 diabetes: a 12-week, randomized, placebo-controlled, Phase 2 study comparing different dose-escalation schemes. Diabetes Obes Metab 25:2805–2814 37311722 10.1111/dom.15168

[73] Karakasis P, Patoulias D, Pamporis K, Stachteas P, Bougioukas KI, Klisic A, Fragakis N, Rizzo M (2023) Safety and efficacy of the new, oral, small-molecule, GLP-1 receptor agonists orforglipron and danuglipron for the treatment of type 2 diabetes and obesity: systematic review and meta-analysis of randomized controlled trials. Metabolism. 10.1016/j.metabol.2023.155710 37852529 10.1016/j.metabol.2023.155710

[74] Che T (2023) *et al.* RCSB PDB - Crystal Structure of a nanobody-stabilized active state of the kappa-opioid receptor. 10.2210/pdb6B73/pdb, accessed on December 08,

[75] Che T et al (2018) Structure of the nanobody-stabilized active state of the kappa opioid receptor. Cell 172:55–67 29307491 10.1016/j.cell.2017.12.011 PMC5802374

[76] Lim C, Priefer R (2022) Pharmacogenomics and pediatric asthmatic medications. J Respir 2:25–43

[77] Papatheodorou A, Makrythanasis P, Kaliakatsos M, Dimakou A, Orfanidou D, Roussos C, Kanavakis E, Tzetis M (2010) Development of novel microarray methodology for the study of mutations in the SERPINA1 and ADRB2 genes-Their association with obstructive pulmonary disease and disseminated bronchiectasis in Greek patients. Clin Biochem 43:43–50 19747908 10.1016/j.clinbiochem.2009.08.026

[78] Hostrup M, Onslev J (2022) The beta2-adrenergic receptor-a re-emerging target to combat obesity and induce leanness? J Physiol 600:1209–1227 34676534 10.1113/JP281819

[79] Dong J-H, Chen X, Cui M, Yu X, Pang Q, Sun J-P (2012) Beta2-adrenergic receptor and astrocyte glucose metabolism. J Mole Neurosci 48:456–463 10.1007/s12031-012-9742-4 22399228

[80] Aránguiz-Urroz P, Canales J, Copaja M, Troncoso R, Vicencio JM, Carrillo C, Lara H, Lavandero S, Díaz-Araya G (2011) Beta2-adrenergic receptor regulates cardiac fibroblast autophagy and collagen degradation. Biochimica et Biophysica Acta (BBA)-Mole Basis Dis 1812:23–31 10.1016/j.bbadis.2010.07.003 20637865

[81] Ishchenko A (2023) *et al.* XFEL beta2 AR structure by ligand exchange from Alprenolol to Timolol. 10.2210/pdb6PS1/pdb, accessed on December 08,

[82] Zimmerman TJ, Kaufman HE (1977) Timolol: a -adrenergic blocking agent for the treatment of glaucoma. Arch Ophthalmol 95:601–604 322648 10.1001/archopht.1977.04450040067008

[83] Martini ML et al (2019) Defining structure-functional selectivity relationships (SFSR) for a class of non-catechol dopamine D1 receptor agonists. J Med Chem 62:3753–3772 30875219 10.1021/acs.jmedchem.9b00351 PMC6688508

[84] Talukdar S et al (2010) GPR120 is an omega-3 fatty acid receptor mediating potent anti-inflammatory and insulin-sensitizing effects. Cell 142:687–698 20813258 10.1016/j.cell.2010.07.041 PMC2956412

[85] Mao C, Xiao P, Tao X, Qin J, He Q, Zhang C, Yu X, Zhang Y, Sun J (2023) Cryo-EM structure of the TUG891 bound GPR120-Gi complex. 10.2210/pdb8ID8/pdb, accessed on December 08,

[86] Mao C et al (2023) Unsaturated bond recognition leads to biased signal in a fatty acid receptor. Science 380:6220 10.1126/science.add6220 36862765

