# Supplementary material for: SpaceGrow: efficient shape-based virtual screening of billion-sized combinatorial fragment spaces
Source: J Comput Aided Mol Des. 2024 Mar 17;38(1):13. doi: 10.1007/s10822-024-00551-7 (PMC10944417; doi:10.1007/s10822-024-00551-7)
Supplement: Supplementary file 1 — (pdf 33490 KB) [file 10822_2024_551_MOESM1_ESM.pdf]

Supplementary Information - SpaceGrow: Efficient Shape-based Virtual Screening of Billion-sized Combinatorial Fragment Spaces

Sophia M. N. Hönig orc,[†,‡] Florian Flachsenberg orc,[†] Christiane Ehrt orc,[‡] Alexander Neumann orc,[†] Robert Schmidt orc,[†] Christian Lemmen orc,[*,†] and Matthias Rarey orc[*,‡]

†*BioSolveIT, An der Ziegelei 79, 53757 Sankt Augustin, Germany*

‡*Universität Hamburg, ZBH - Center for Bioinformatics, Bundesstraße 43, 20146 Hamburg, Germany*

E-mail: christian.lemmen@biosolveit.de; matthias.rarey@uni-hamburg.de

Conformer Generation As a sanity check, the performance of SpaceGrow to retrieve molecules in different conformations was evaluated on the 6x10^4 eXplore S subsample. For this purpose, one hundred enumerated molecules from each reaction were randomly sampled and utilized as MOIs for SpaceGrow. This makes a total of 300 requests for searching in the space. These molecules were directly enumerated from the database. Thus, they have the conformations derived from the database fragments. Additionally, conformers for these molecules were generated by using RDKit[1], Conformator[2] and 3D-generator[3]. Since the implementation for conformer generation in RDKit has different standards for bond lengths and a different torsion profile, we generated 100 conformers per molecule and picked conformer with the lowest RMSD to the conformation from the database for each. Conformator

and 3D-generator share standards similar to our chemistry model, so only one conformation was generated and later used for the experiment.

Conformer Validation All MOIs randomly sampled from the database for amide coupling and substitution reactions were found at the best rank by SpaceGrow. Of the hundred MOIs for ring-closing reactions, only six molecules were not found at the best rank. However, in all six cases, the best ranked molecule had a score equal to the score of the MOI. In case of an equal score, SpaceGrow results are ranked by an internal molecule identifier explaining the shift of the MOI in its rank.

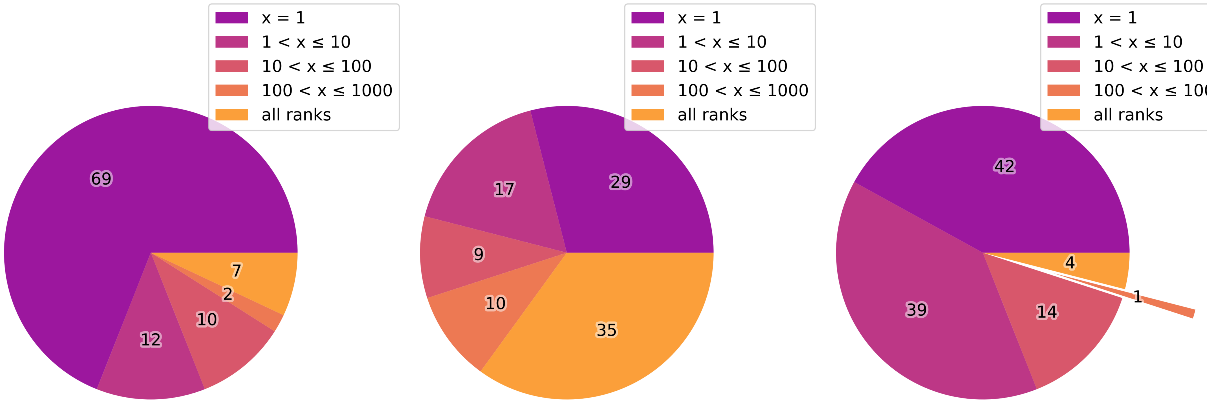

(a) Amide coupling (b) Substitution (c) Ring-closing

Figure 1: Retrieval of MOIs with RDKit conformers.

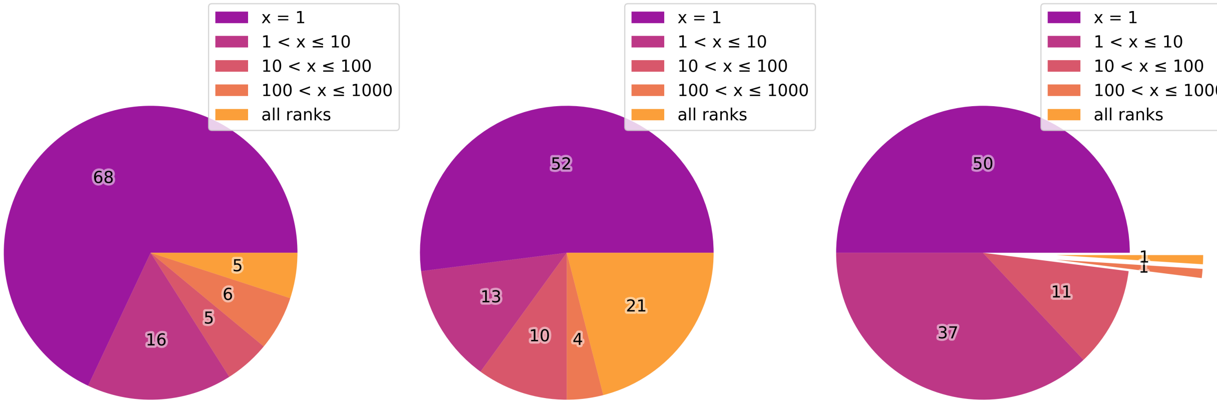

(a) Amide coupling (b) Substitution (c) Ring-closing

Figure 2: Retrieval of MOIs with Conformator conformers.

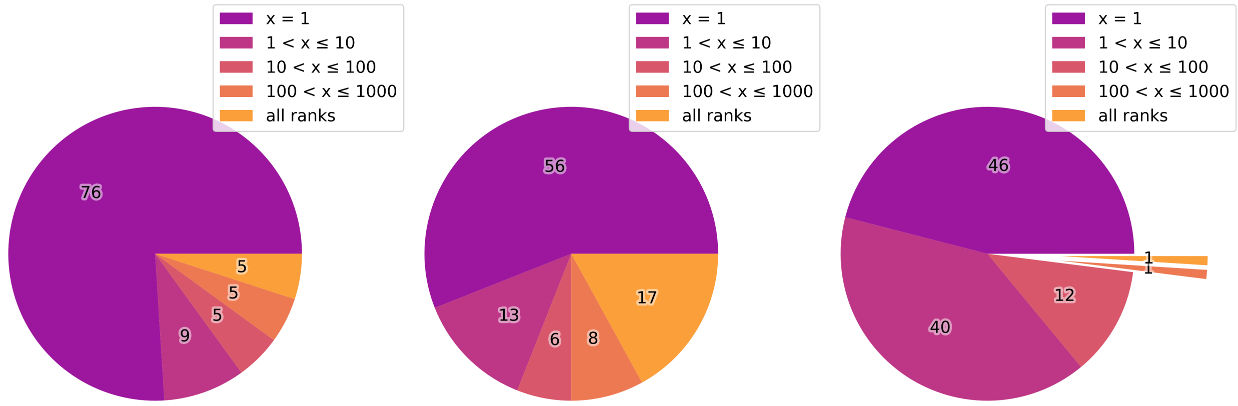

(a) Amide coupling (b) Substitution (c) Ring-closing

Figure 3: Retrieval of MOIs with 3D-generator conformers.

Analyzing the retrieval for the conformers generated by RDKit, Conformator and 3D-generator, SpaceGrow achieved a retrievel of over 80 % among the top ten ranks for the MOIs of both amide coupling and ring-closing reactions. The retrieval of molecules generated from substitution reactions seems more challenging. For the conformers of 3D-generator and Conformator, SpaceGrow found 69 % and 65 % of the MOIs among the first ten ranks, respectively. For the conformers generated by RDKit, only 46 % of the MOIs were found among the first ten ranks. Detailed results are depicted in Figures 1, 2 and 3.

It remains unclear why products of the substitution reactions seem harder to retrieve from the space. Having a closer look at the reactions[4], one possible reason might be a higher flexibility at the attachment point at which the fragments are combined. This would make the task of finding a proper exit bond to cut at the MOI more difficult.

Tool Comparison on Validation Space and Library The tools SpaceGrow and LS-align[5] were tested on the validation space and library, respectively. Here, it is analyzed whether the tools consider the same binding site alignment-based superpositions difficult to reproduce. Thus, for each tool, the ten ensembles were compared, for which the reproduced pose of the homologous ligand showed the highest RMSD. Four common ensembles were found within the ten highest RMSD results of both tools. These are shown in Figure 4.

Figures 4a and 4b show that a possible reason for the high RMSD values that occurred reproducing the superpositions of the ligands with the three-letter codes AXI from PDB-ID 4AG8 and FUN from PDB-ID 2XN5 are the ring systems. Note that the native superpositions were derived from a sequence-based alignment of the protein pockets so that the bound ligands were superposed in their native binding mode. For these two examples, the ring systems and the polar atoms of the MOIs and the homologous ligands are not properly aligned in the native superpositions. For this reason, the tools might have generated poses that deviate so strongly from the native pose. For the ligand with the three-letter code AXI, SpaceGrow generated a pose with an RMSD of 10.1 Å. The pose generated by LS-align had an RMSD of 10.2 Å. For the superpositions of the ligand with the three-letter code FUN generated by SpaceGrow the RMSD value was 6.5 Å. LS-align generated a pose with an RMSD of 6.9 Å.

Reproducing superposition of the ligands with the three-letter codes AGF and CIT PDB-IDs 8CPA and 3KGQ, respectively, is almost impossible, as Figure 4c shows. Since the ligand with the three-letter code CIT is much smaller than the ligand with the three-letter code AGF, it is not a good template for a superposition and probably should have been sorted out in the initial filtering for suitable MOIs. The pose generated by SpaceGrow showed an RMSD of 6.8 Å, while the aligned ligands of LS-align had an RMSD of 11.1 Å.

Even though the sizes of the ligand with the three-letter code 6J3 with the PDB-ID 6GNR and the homologous ligand with the three-letter code 8PF with the PDB-ID 6GON are similar, as Figure 4d reveals, there are almost no key features of the two molecules. Apart from a carboxylic acid moiety, the two molecules have no ring systems or polar atoms in common. This molecular type is a very challenging task to superposition methods, making the homologous pose almost unpredictable by only knowing the MOI pose. The pose generated by SpaceGrow had an RMSD value of 5.9 Å. LS-align was not able to generate a pose for the ligand with the three-letter code 8PF.

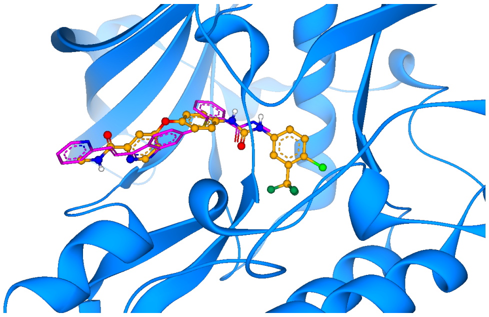

(a) Reference ligand BAX in the protein structure with PDB-ID 4ASD with homologous ligand AXI from the structure with PDB-ID 4AG8.

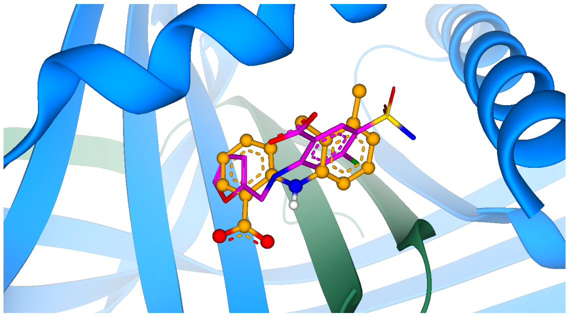

(b) Reference ligand ID8 in protein structure 2XN3 with homologous ligand FUN from structure 2XN5.

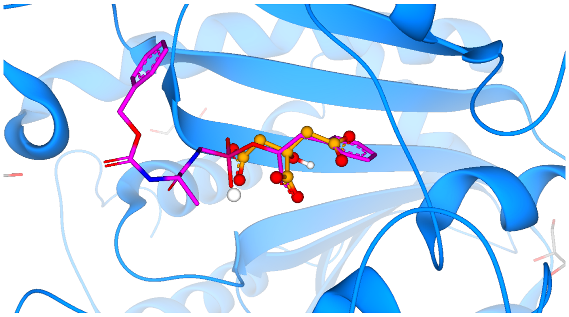

(c) Reference ligand CIT in protein structure 3KGQ with homologous ligand AGF from structure 8CPA.

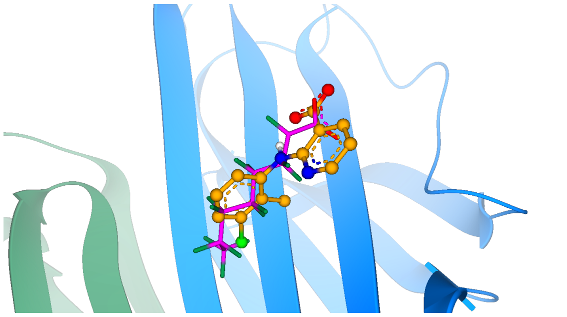

(d) Reference ligand 6J3 in protein structure 6GNR with homologous ligand 8PF from structure 6GON.

Figure 4: Ensembles with superpositions that were difficult for SpaceGrow and LS-align to reproduce. The reference ligand (MOI) is shown in orange. The homologous ligand is shown in pink. Ligands are shown in the protein structure of the reference ligand used for sequence-based site alignment.

Comparison of Results from Spaces of Different Size Additional results for the analysis of searching the 160 knwon active MOIs within the 6x10^9 eXplore 2C and the 6x10^4 eXplore S space. After HYDE optimization and scoring, the top-ranked results were classified by their ligand efficiency.

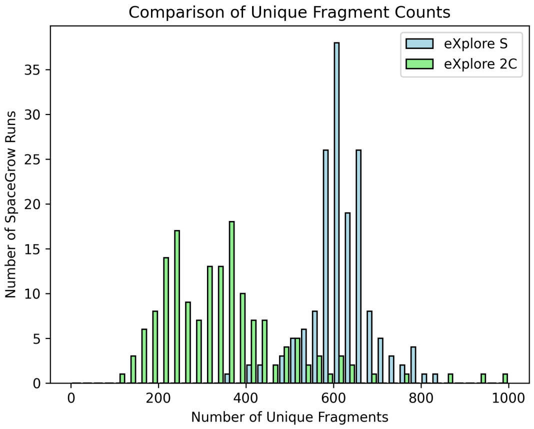

(a) Unique fragment count for top thousand results of 160 SpaceGrow runs.

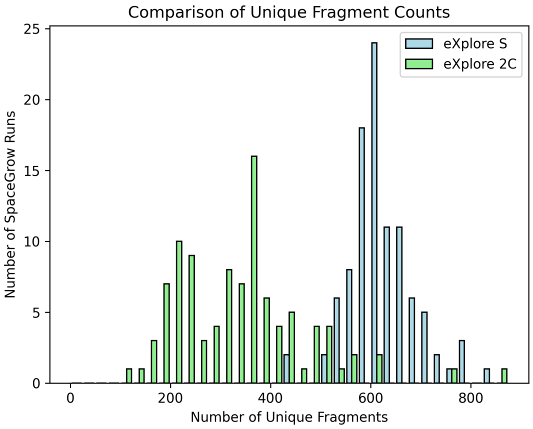

(b) Unique fragment count top hundred results of 160 SpaceGrow runs.

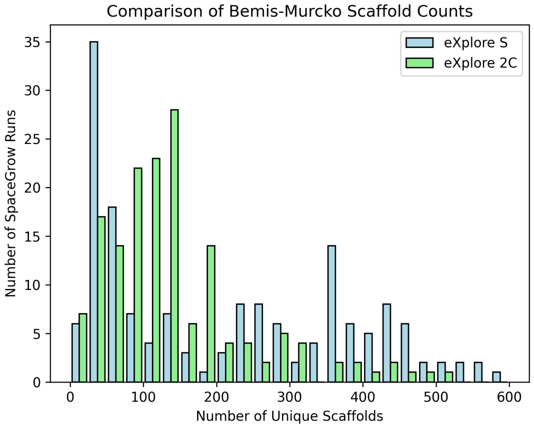

(c) Bemis-Murcko scaffold count for top thousand results of 160 SpaceGrow runs.

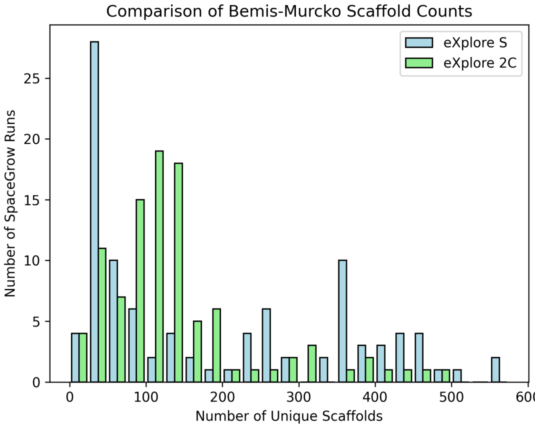

(d) Bemis-Murcko scaffold count for top hundred results of 160 SpaceGrow runs.

Figure 5: Counts for the number of unique fragments and unique Bemis-Murcko scaffolds within the top thousand and top hundred SpaceGrow results for searching the 160 MOIs in eXplore S andeXplore 2C, respectively.

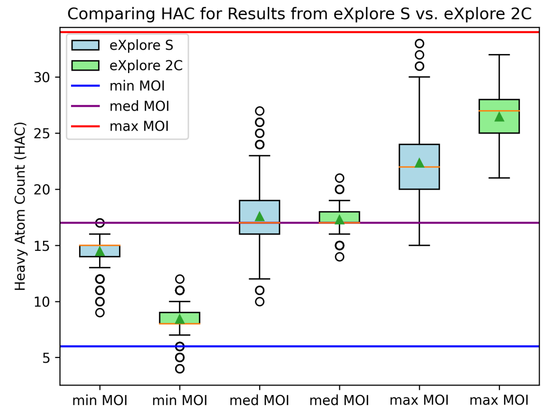

(a) HAC comparison for top thousand results

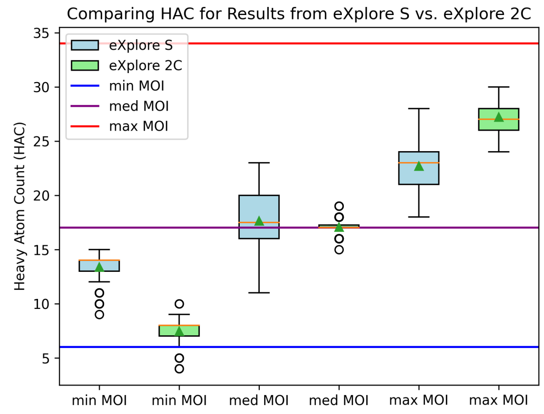

(b) HAC comparison for top hundred results

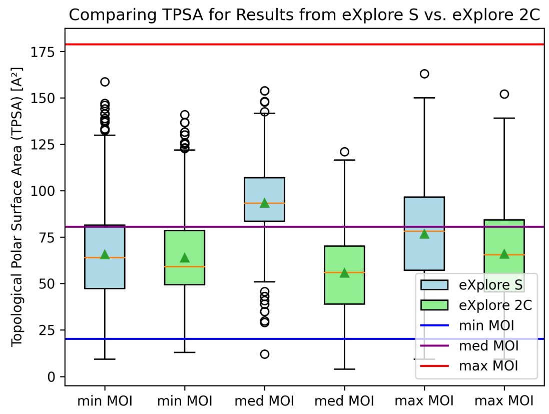

(c) TPSA comparison for top thousand results

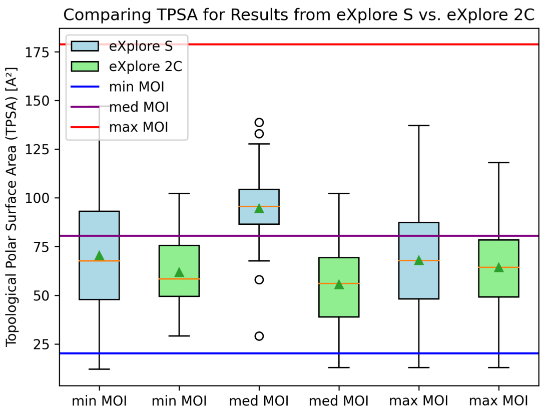

(d) TPSA comparison for top hundred results

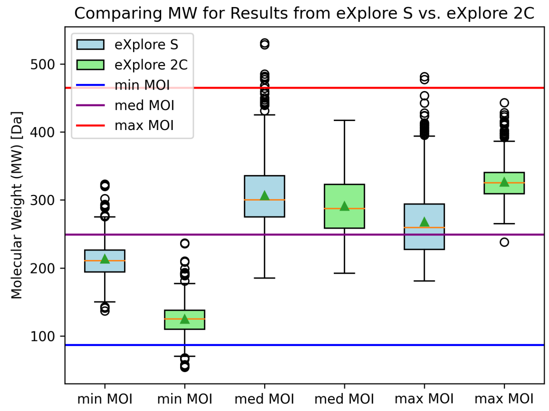

(e) MW comparison for top thousand results

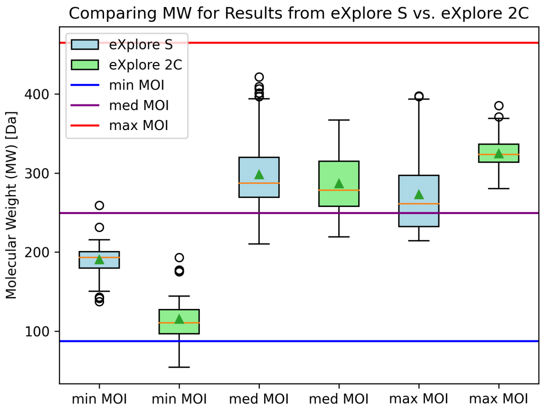

(f) MW comparison for top hundred results

Figure 6: Comparison of the properties of the top thousand or top hundred SpaceGrow results for the MOI with the minimum (min), median (med) and maximum (max) property value for eXplore S and eXplore 2C, respectively. Horizontal lines show the property of the min, max and med MOI. Each boxplot relates to the resuls of the search for one of the MOIs. For each boxplot, the median is given by the orange line and the average by the green triangle.

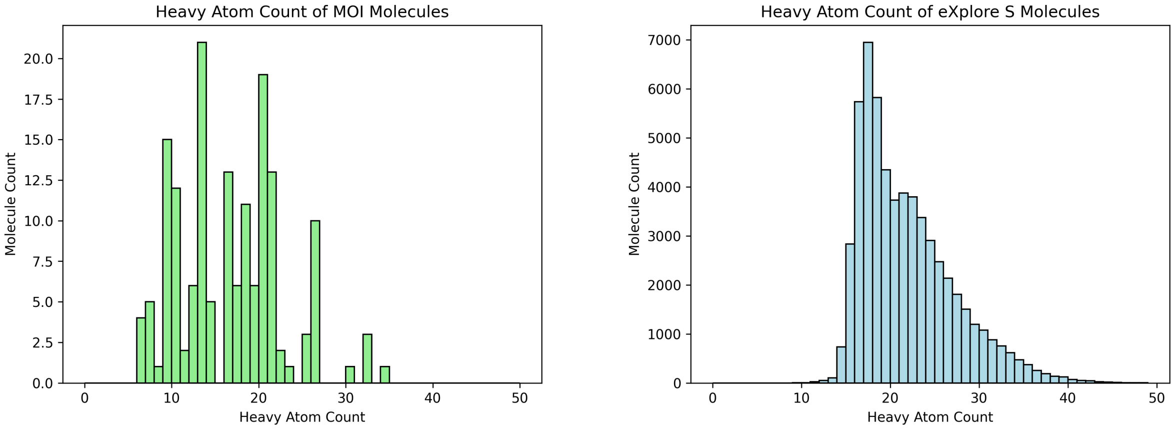

(a) (b)

Figure 7: Comparison of heavy atom count (HAC) distribution between (a) the 160 MOI molecules and (b) the molecules of the enumerated eXplore S space.

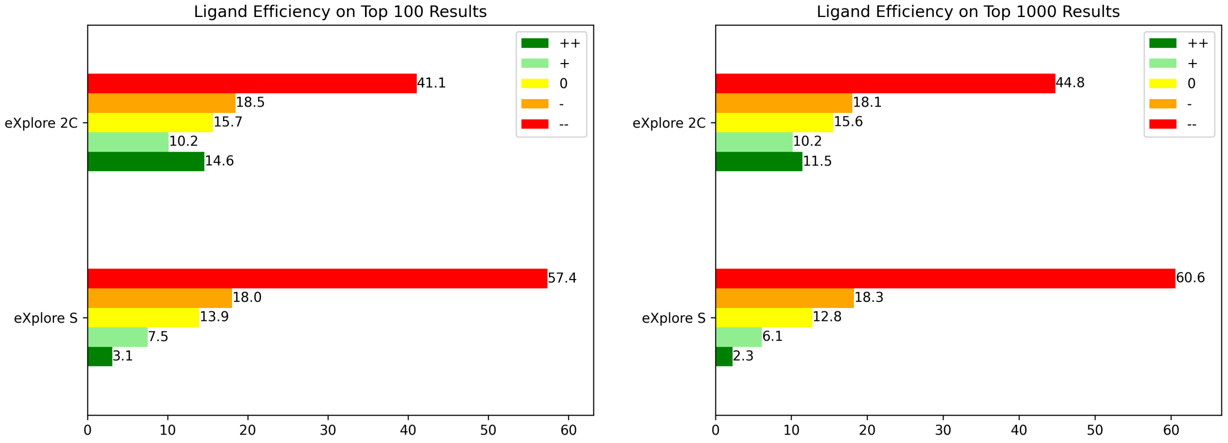

(a) Top hundred SpaceGrow results (b) Top thousand SpaceGrow results

Figure 8: Ligand efficiency classes predicted by HYDE (a) for the top hundred and (b) for the top thousand result molecules for each search of the 160 MOIs on the $6x10^4$ eXplore S and the $6x10^9$ eXplore 2C space. Bars provide the percentage of molecules rated by the underlying ligand efficiency class. Dark green (++) is rated as best and red (−−) as least efficient.

Run Time on $6x10^4$ eXplore S Space Additional figure of the run time evaluation for the search of the 160 known active MOIs on the $6x10^4$ eXplore S space.

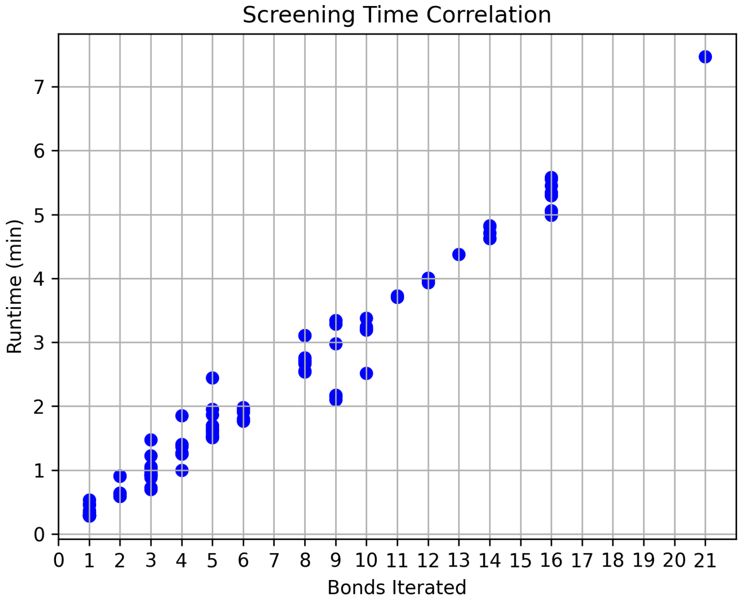

Figure 9: Correlation of number of bonds in the MOI iterated and run time of the search in the eXplore S space.

Mining for Potential Binders Additional information on the SpaceGrow results for the MOIs of the four GPCRs within the structures with the PDB-IDs 7LCK,[6,7] 6B73,[8,9] 6PS1,[10,11] and 8ID8[12,13]. In the following tables, the compounds of the top ten results after filtering for affinity and Tanimoto similarity based on the Morgan Fingerprint implementation with radius four from RDKit[1] are provided with their estimated affinity lower bound and their tanimoto similarity to the MOI.

Table 1: Additional Results for the structure of the human glucagon-like peptide-1 receptor (7LCK) with danuglipron as MOI (light blue) and HYDE optimized SpaceGrow results (orange). Aff. is the estimated affinity lower bound in nM and Tan. is the Tanimoto similarity to the MOI.

**3D Alignment**	**Compound Structure**	**Aff.**	**Tan.**
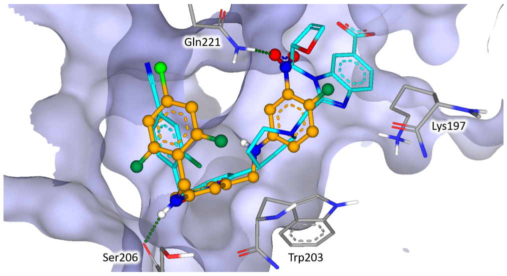	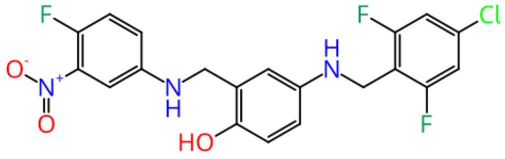	0.70	0.12
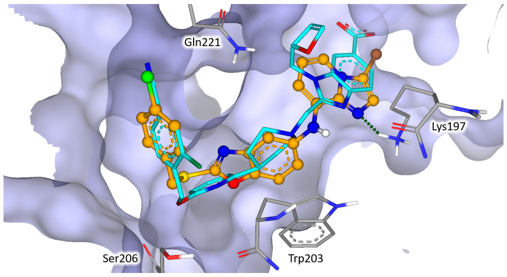	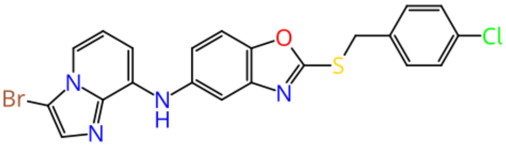	0.11	0.13
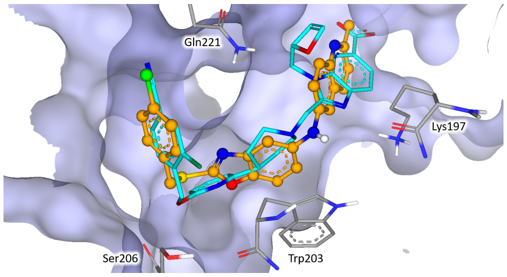	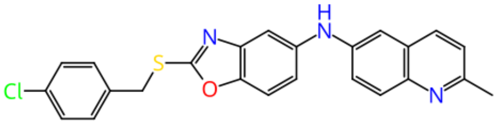	0.17	0.14

Continued on next page

Table 1 – continued from previous page

3D Alignment	Compound Structure	Aff.	Tan.
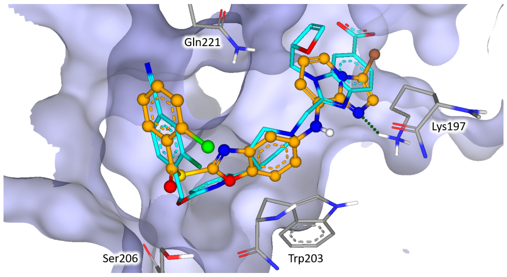	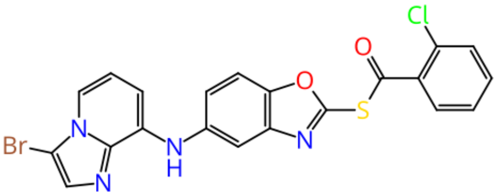	0.69	0.14
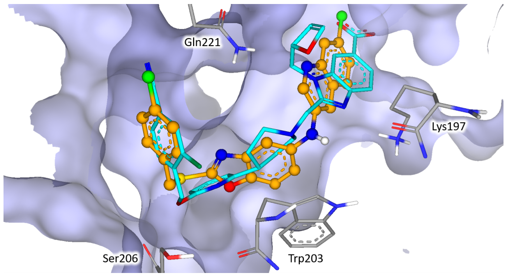	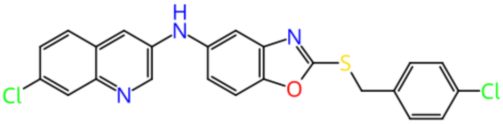	0.18	0.13
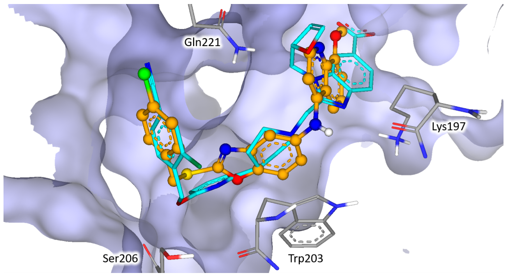	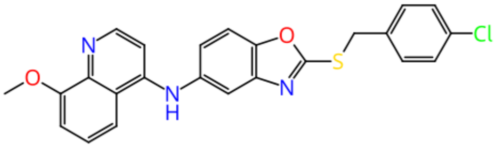	0.29	0.13
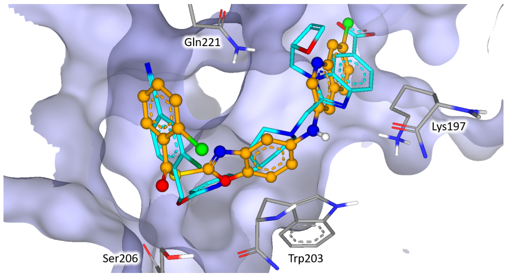	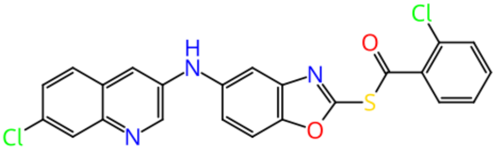	0.21	0.14

Table 2: Additional Results for the structure of the kappa-opioid receptor (6B73) with an apomorphin derivate as MOI (light blue) and HYDE optimized SpaceGrow results (orange). Aff. is the estimated affinity lower bound in nM and Tan. is the Tanimoto similarity to the MOI.

**3D Alignment**	**Compound Structure**	**Aff.**	**Tan.**
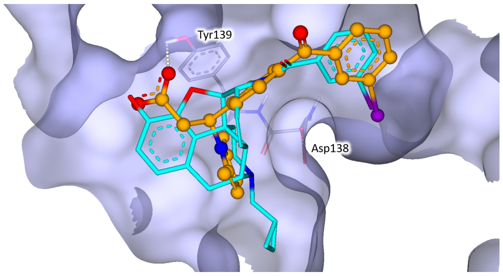	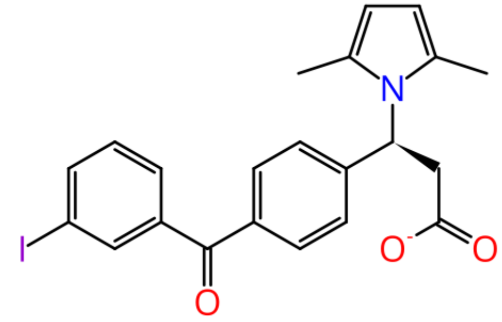	1.44	0.17
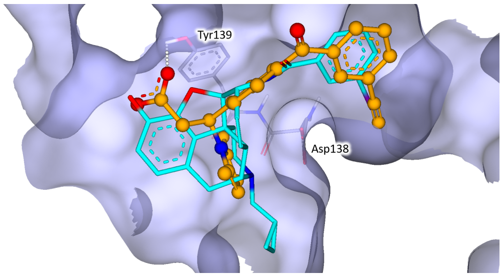	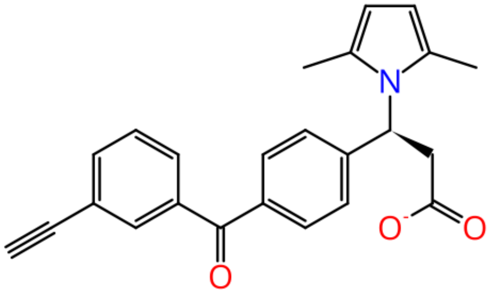	1.02	0.12
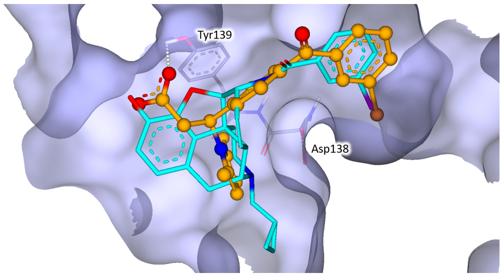	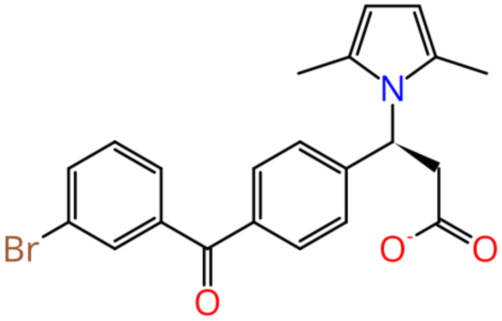	1.21	0.13

Continued on next page

3D Alignment	Compound Structure	Aff.	Tan.
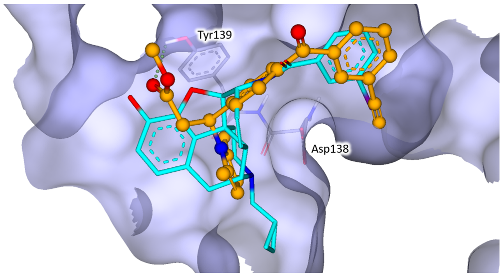	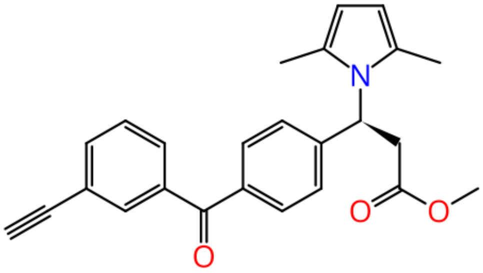	2.38	0.12
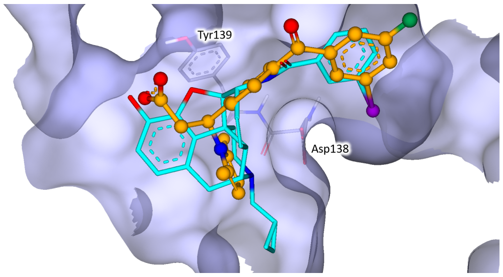	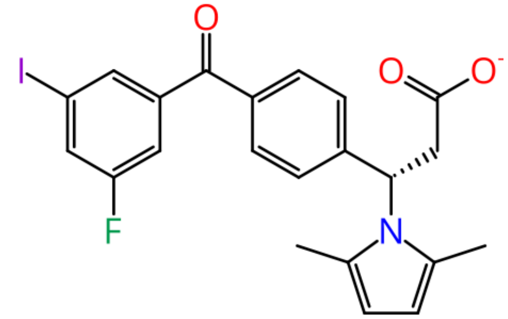	1.04	0.13
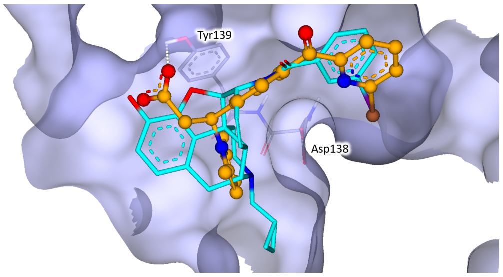	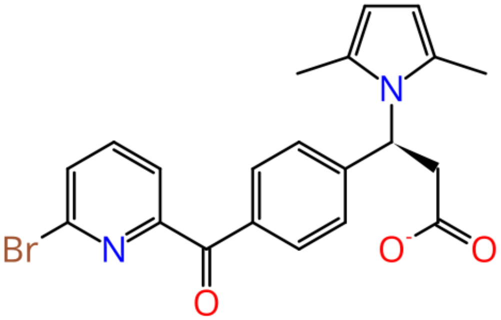	2.67	0.12
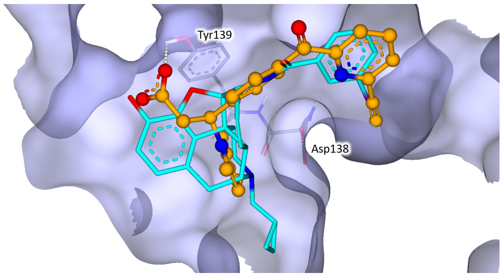	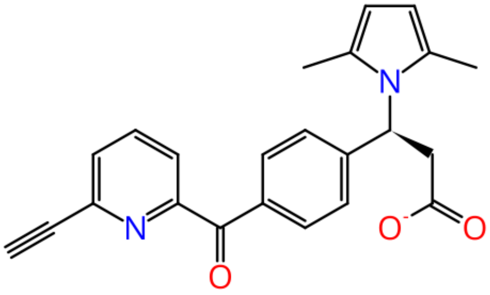	2.23	0.11

Continued on next page

Table 2 – continued from previous page

3D Alignment	Compound Structure	Aff.	Tan.
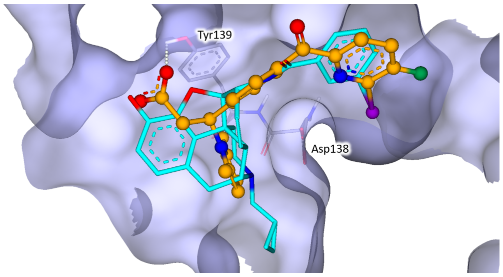	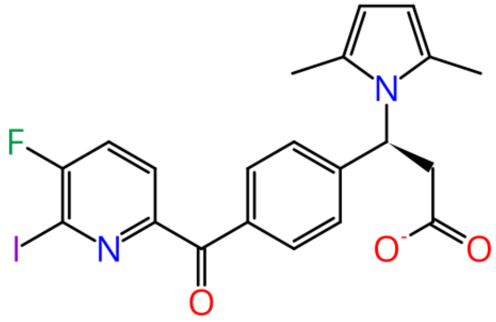	1.21	0.12
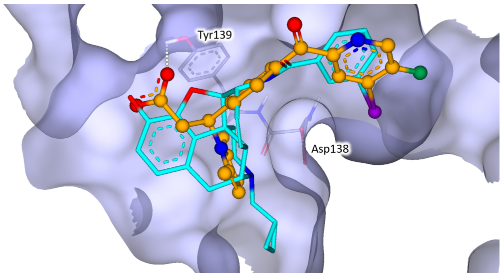	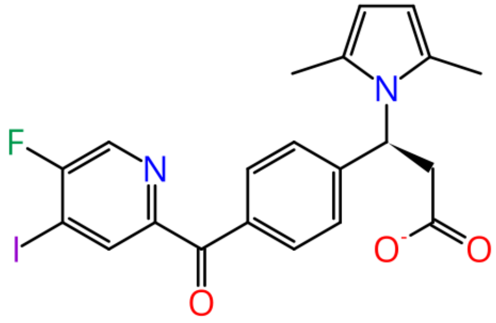	1.31	0.12

Table 3: Additional Results for the structure of the β-2 adrenergic receptor (6PS1) with timolol as MOI (light blue) and HYDE optimized SpaceGrow results (orange). Aff. is the estimated affinity lower bound in nM and Tan. is the Tanimoto similarity to the MOI.

3D Alignment	Compound Structure	Aff.	Tan.
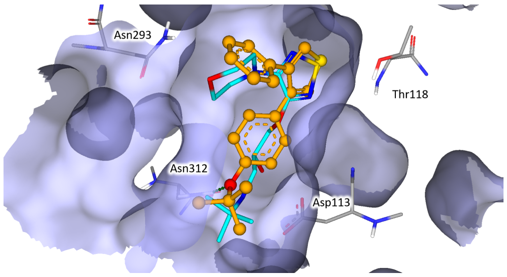	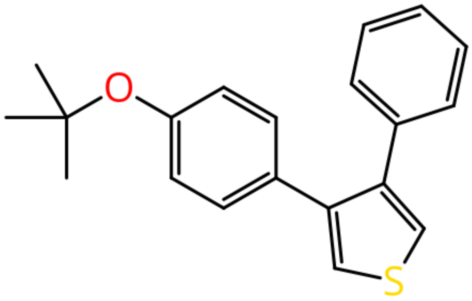	0.09	0.07

Continued on next page

Table 3 – continued from previous page

3D Alignment	Compound Structure	Aff.	Tan.
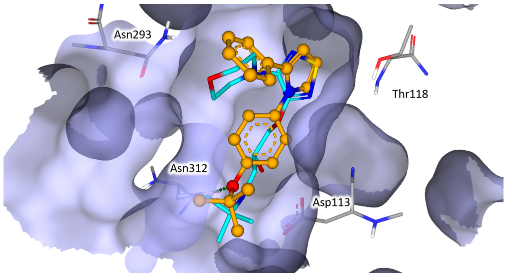	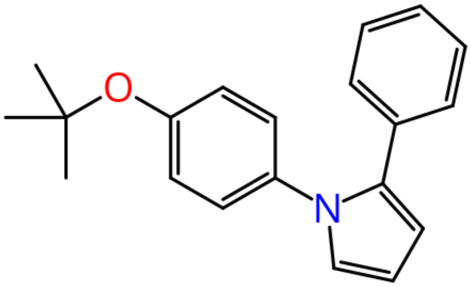	0.27	0.07
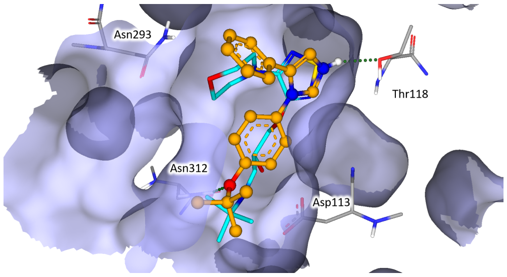	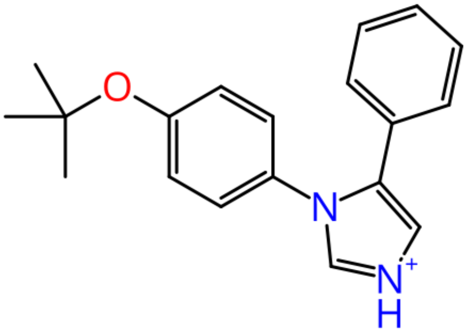	0.77	0.07
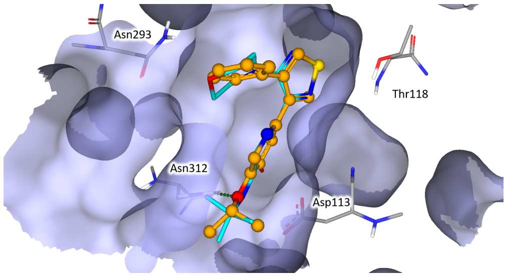	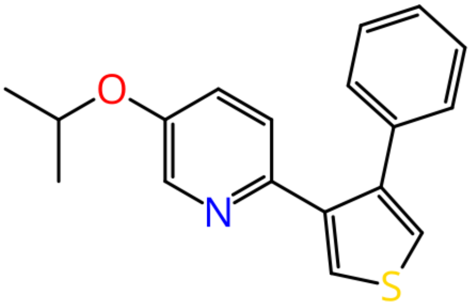	1.95	0.05
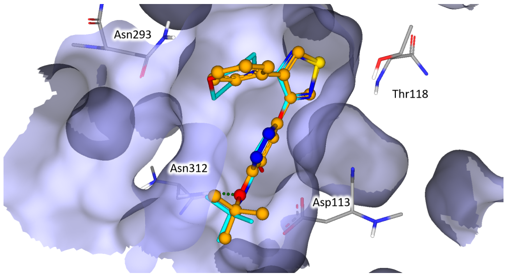	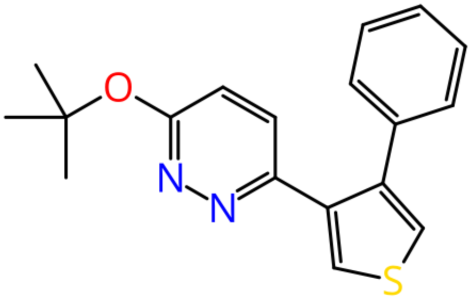	0.81	0.09

Continued on next page

Table 3 – continued from previous page

3D Alignment	Compound Structure	Aff.	Tan.
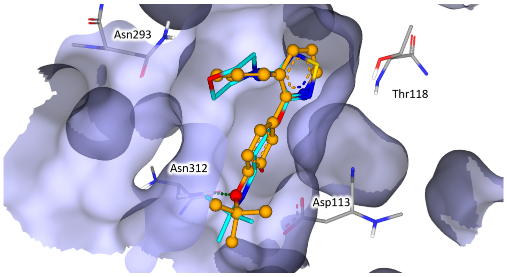	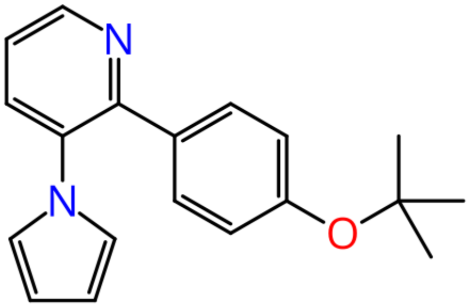	1.48	0.08
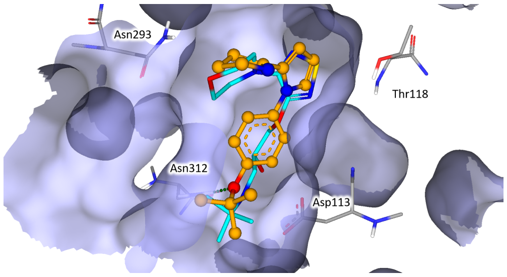	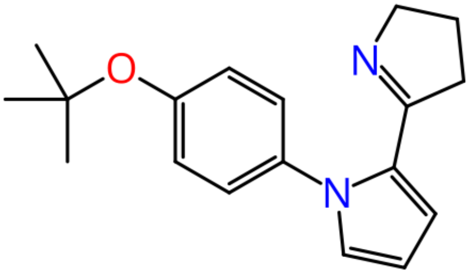	3.42	0.10
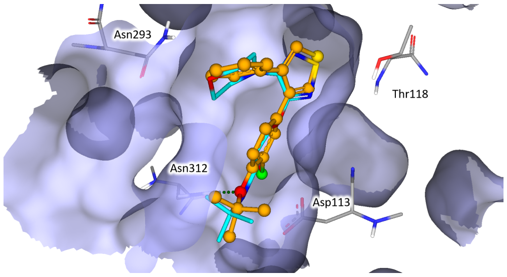	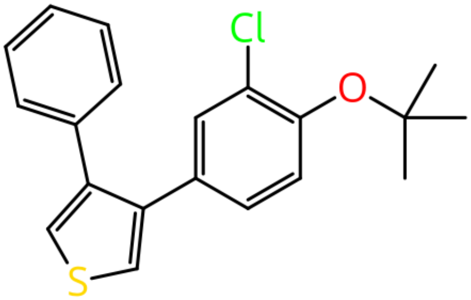	0.86	0.07
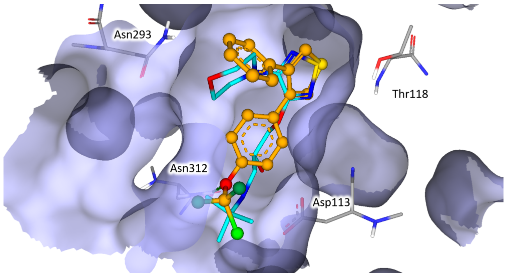	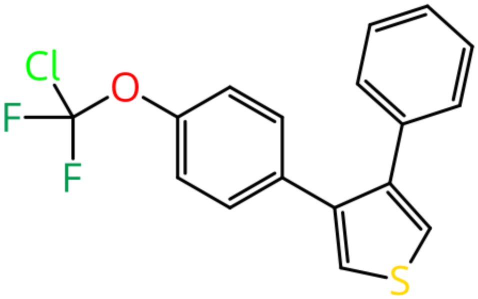	1.21	0.03

Table 4: Additional Results for the structure of the Free Fatty acid receptor 4 (FFAR4/GPR120) (8ID8) with TG891 as MOI (light blue) and HYDE optimized SpaceGrow results (orange). Aff. is the estimated affinity lower bound in nM and Tan. is the Tanimoto similarity to the MOI.

**3D Alignment**	**Compound Structure**	**Aff.**	**Tan.**
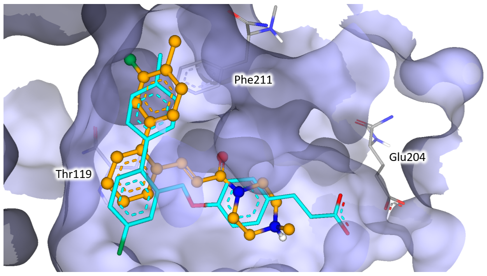	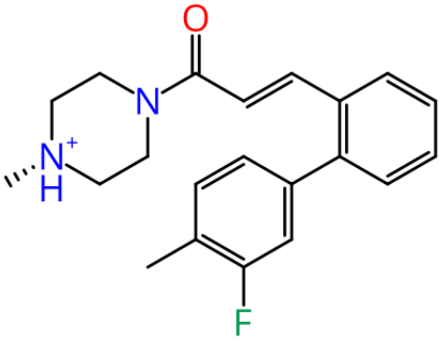	1.09	0.15
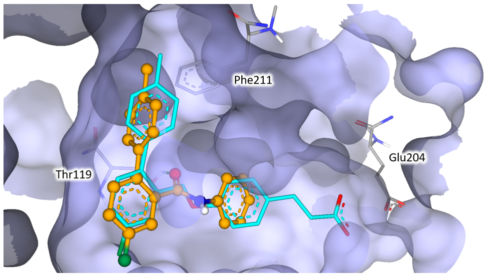	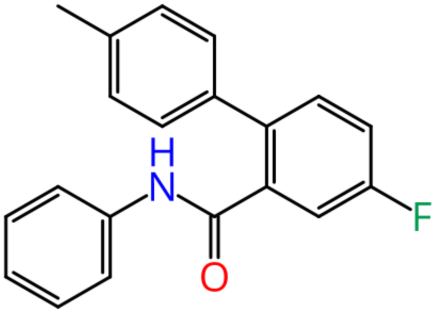	2.77	0.34
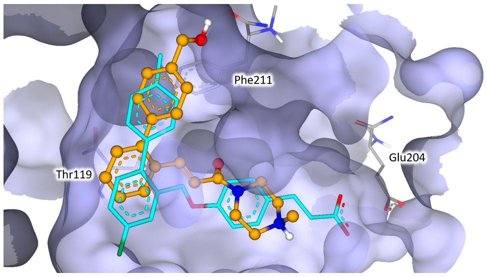	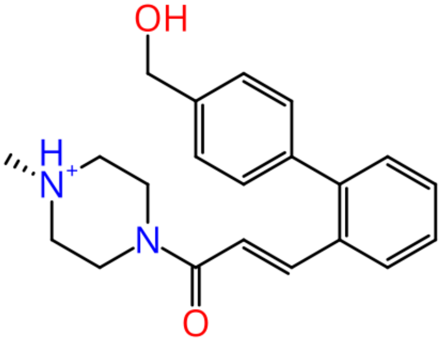	1.37	0.18

Continued on next page

Table 4 – continued from previous page

3D Alignment	Compound Structure	Aff.	Tan.
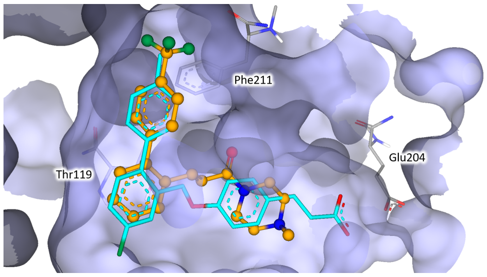	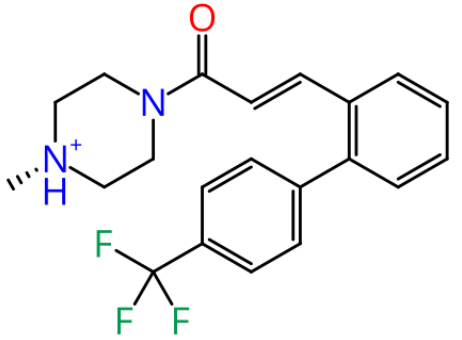	2.91	0.15
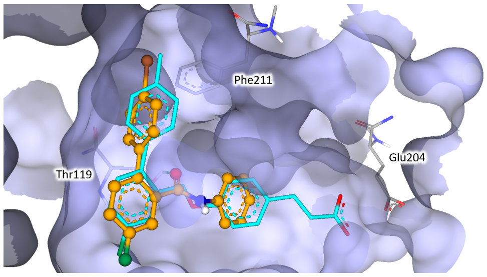	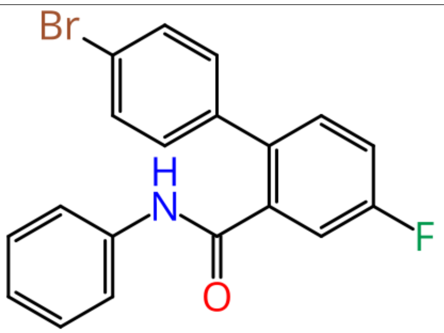	2.32	0.26
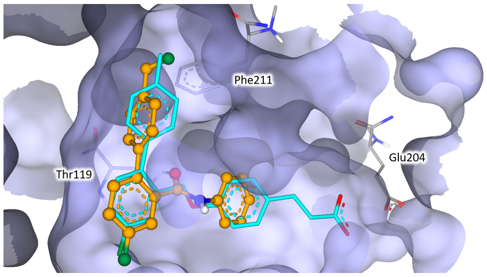	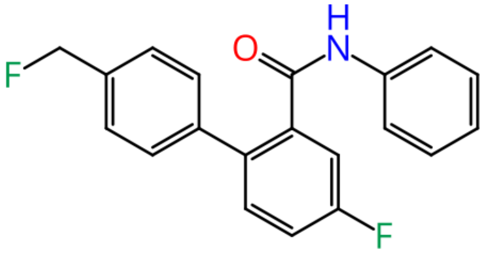	1.74	0.29
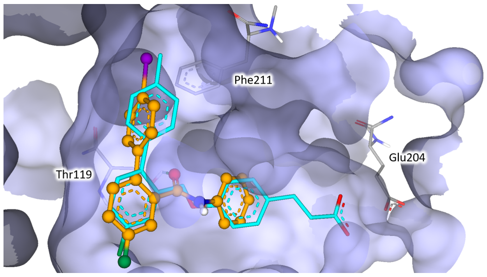	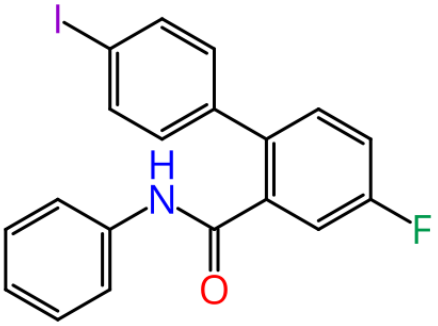	1.26	0.26

Continued on next page

Table 4 – continued from previous page

3D Alignment	Compound Structure	Aff.	Tan.
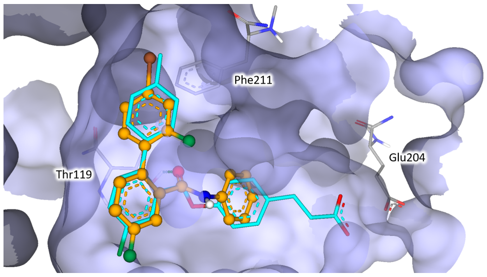	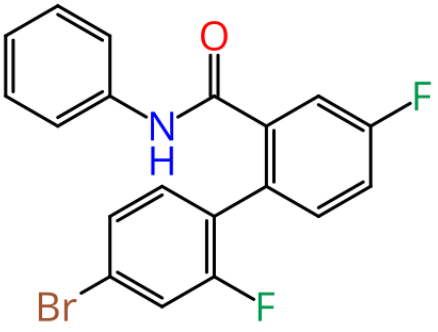	0.73	0.22
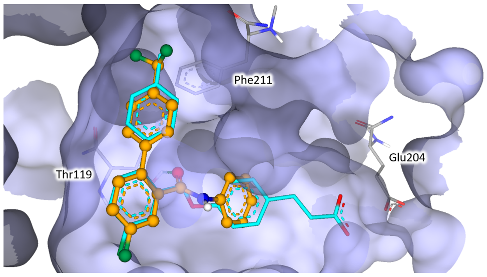	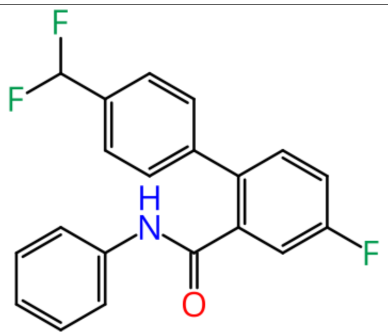	0.39	0.25

References

(1) Landrum, G. . RDKit. https://www.rdkit.org/, accessed on November 03, 2023.

(2) Friedrich, N.-O.; Flachsenberg, F.; Meyder, A.; Sommer, K.; Kirchmair, J.; Rarey, M. Conformator: a novel method for the generation of conformer ensembles. *Journal of Chemical Information and Modeling* **2019**, *59*, 731–742.

(3) BioSolveIT GmbH KNIME interfaces - Drug discovery workflows - BioSolveIT. https://www.biosolveit.de/KNIME, accessed on November 03, 2023.

(4) BioSolveIT GmbH eXplore Cookbook. https://www.biosolveit.de/infiniSee/cookbook, accessed on January 22, 2024.

(5) Hu, J.; Liu, Z.; Yu, D.-J.; Zhang, Y. LS-align: an atom-level, flexible ligand structural

alignment algorithm for high-throughput virtual screening. *Bioinformatics* **2018**, *34*, 2209–2218.

(6) Belousoff, M.; Johnson, R.; Drulyte, I.; Yu, L.; Kotecha, R., A.and Danev; Wootten, D.; Zhang, X.; Sexton, P. RCSB PDB - PF 06882961 bound to the glucagon-like peptide-1 receptor (GLP-1R). https://doi.org/10.2210/pdb7LCK/pdb, accessed on December 08, 2023.

(7) Zhang, X.; Johnson, R. M.; Drulyte, I.; Yu, L.; Kotecha, A.; Danev, R.; Wootten, D.; Sexton, P. M.; Belousoff, M. J. Evolving cryo-EM structural approaches for GPCR drug discovery. *Structure* **2021**, *29*, 963–974.

(8) Che, T. et al. RCSB PDB - Crystal Structure of a nanobody-stabilized active state of the kappa-opioid receptor. https://doi.org/10.2210/pdb6B73/pdb, accessed on December 08, 2023.

(9) Che, T.; Majumdar, S.; Zaidi, S. A.; Ondachi, P.; McCorvy, J. D.; Wang, S.; Mosier, P. D.; Uprety, R.; Vardy, E.; Krumm, B. E.; others Structure of the nanobody-stabilized active state of the kappa opioid receptor. *Cell* **2018**, *172*, 55–67.

(10) Ishchenko, A. et al. XFEL beta2 AR structure by ligand exchange from Alprenolol to Timolol. https://doi.org/10.2210/pdb6PS1/pdb, accessed on December 08, 2023.

(11) Liu, Z.; Singh, S. B.; Zheng, Y.; Lindblom, P.; Tice, C.; Dong, C.; Zhuang, L.; Zhao, Y.; Kruk, B. A.; Lala, D.; others Discovery of Potent Inhibitors of 11β-Hydroxysteroid Dehydrogenase Type 1 Using a Novel Growth-Based Protocol of in Silico Screening and Optimization in CONTOUR. *Journal of Chemical Information and Modeling* **2019**, *59*, 3422–3436.

(12) Mao, C.; Xiao, P.; Tao, X.; Qin, J.; He, Q.; Zhang, C.; Yu, X.; Zhang, Y.; Sun, J. Cryo-EM structure of the TUG891 bound GPR120-Gi complex. https://doi.org/10.2210/pdb8ID8/pdb, accessed on December 08, 2023.

(13) Mao, C.; Xiao, P.; Tao, X.-N.; Qin, J.; He, Q.-T.; Zhang, C.; Guo, S.-C.; Du, Y.-Q.; Chen, L.-N.; Shen, D.-D.; others Unsaturated bond recognition leads to biased signal in a fatty acid receptor. *Science* **2023**, *380*, eadd6220.
